# Study of fluconazole drug behavior in deep eutectic solvents: thermodynamic properties, solubility measurement, and fluorescence spectroscopy

**DOI:** 10.1039/d4ra09043h

**Published:** 2025-04-09

**Authors:** Hadi Azad, Hemayat Shekaari, Fariba Ghaffari, Masumeh Mokhtarpour, Mohammad Bagheri Hokm Abad

**Affiliations:** a Department of Physical Chemistry, University of Tabriz Tabriz Iran hemayatt@yahoo.com +98-4133340191 +98-4133393094

## Abstract

Fluconazole is a crucial antifungal medication with a broad spectrum of activity against various fungal infections. This study thermodynamic properties, solubility measurements and spectrofluorometric method were used for investigating the interactions between fluconazole (FCZ) and deep eutectic solvents (DESs). Five choline chloride-based deep eutectic solvents (DESs) were synthesized. Each DES was prepared by combining choline chloride (a hydrogen bond acceptor, HBA) with a different hydrogen bond donor (HBD): oxalic acid (OX), malonic acid (MA), ethylene glycol (EG), glycerol (G), or urea (U). Subsequently, the interactions between fluconazole (FCZ) and these synthesized DESs were investigated using fluorescence spectroscopy at a temperature of 298.15 K. Fluorescence spectroscopy revealed a strong interaction between fluconazole (FCZ) and deep eutectic solvents (DESs). This was evident from the significant quenching of FCZ's intrinsic fluorescence upon DES addition. The association constant and binding sites were determined. Among the tested DESs, the choline chloride-oxalic acid mixture exhibited the strongest interaction with FCZ. Furthermore, the solubility of FCZ in DES-water mixtures studied at a temperature range of (298.15 to 313.15) K was found to increase with increasing DES concentration. The solubility data were accurately fitted using the e-NRTL and Wilson thermodynamic models. To gain deeper insights, conductor-like screening model (COSMO) calculations were performed on the studied systems. The obtained surface cavity volume and dielectric solvation energy provide valuable information about the intermolecular interactions. Finally, thermodynamic analysis using Gibbs and van't Hoff equations indicated that the dissolution of FCZ in these systems is an endothermic process.

## Abstract 2

Fluorescence and solubility analysis of fluconazole in bio-based deep eutectic solvents (DESs): experimental and modeling approach (e-NRTL, Wilson).

## Graphical abstract

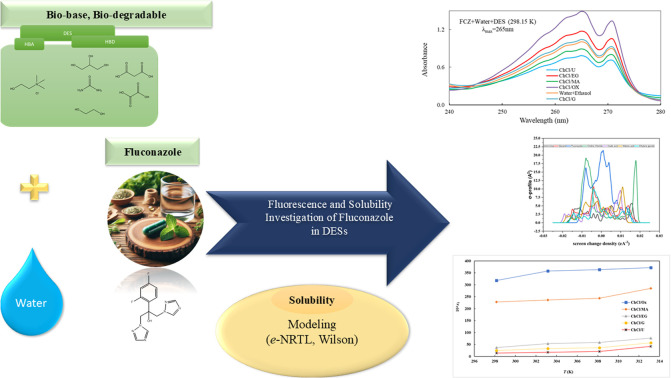

## 1. Introduction

A significant challenge in modern pharmaceutical development lies in overcoming the poor solubility, permeability, and bioavailability of numerous drug candidates, which ultimately hinders their therapeutic efficacy. These limitations often stem from suboptimal pharmacokinetic profiles. To address these issues, deep eutectic solvents (DESs) have emerged as a promising class of solvents with considerable potential in drug delivery formulations. Deep eutectic solvents (DESs) are defined as mixtures of two or more components, typically comprising a hydrogen bond acceptor (HBA) and a hydrogen bond donor (HBD). They exhibit a melting point significantly lower than that of the individual components, a phenomenon attributed to the formation of extensive hydrogen bonding networks among the constituent molecules. This characteristic results in a notable depression of the freezing point. DESs have gained considerable attention as sustainable alternatives to conventional organic solvents and ionic liquids, owing to their inherent environmental advantages.^1–5^

These include their facile preparation from readily available and often biocompatible components, their biodegradability, and their negligible volatility. While their industrial applications are rapidly expanding, the exploration of DESs in pharmaceutical settings is still in its nascent stages. Among the diverse array of DESs, those based on choline chloride (ChCl) as the HBA have emerged as particularly attractive candidates for pharmaceutical applications. Choline chloride, a naturally occurring compound, possesses inherent biocompatibility and has demonstrated promising properties in various preclinical and clinical studies. The versatility of ChCl-based DESs stems from the wide range of HBDs that can be employed, enabling the fine-tuning of their physicochemical properties to suit specific drug delivery requirements.^6–8^ This is due to their unique properties such as low flammability, chemical stability, and low toxicity, which lead to the use of these kinds of DESs in a wide variety of processes in pharmaceutical science.^9,10^

In recent years, these solvents especially DESs based on ChCl have been considered as solvent to improve the solubility of low water-soluble drugs. In this regard, many studies have been done. But to understand and gain deep insight into permeability, bioavailability, or solubility processes, it is essential to have knowledge of interaction mechanisms of drugs and pharmaceutical dynamics. For example, the interaction mechanisms of some drugs with bovine serum albumin (BSA) have been investigated with different methods. Huang *et al.*^11^ studied the interaction of streptomycin sulfate and BSA using flow-injection analysis. Kamat^12^ examined the interaction between fluoroquinolones and bovine serum album. Kandagal *et al.*^13^ had investigated the binding mechanism of an anticancer drug with human serum albumin. Wei *et al.*^14^ studied the association behaviors between biliverdin and bovine serum albumin by fluorescence spectroscopy.

Numerous studies have demonstrated a strong correlation between drug–protein binding interactions and key pharmacokinetic parameters, including apparent volume of distribution and elimination rate.^6,7,15^ These interactions significantly influence the transport and distribution of drugs within the organism, thereby impacting their therapeutic efficacy and potential for adverse reactions. While extensive research has focused on drug–serum albumin interactions, a notable gap exists in the literature regarding the investigation of drug interactions with deep eutectic solvents (DESs) utilizing fluorescence spectroscopy.^16,17^

In the present study, fluconazole was selected as a drug to with five different choline chloride-based DESs made from mixtures of ChCl as hydrogen bond acceptor and oxalic acid (OX), malonic acid (MA), ethylene glycol (EG), glycerol (G) and urea (U) as hydrogen bond donor with specific molar ratio at 298.15 K.

This study investigates the solubility and drug–solvent interactions of fluconazole (FCZ) in a series of choline chloride-based deep eutectic solvents (DESs). The selected DESs, composed of choline chloride (ChCl) as a hydrogen bond acceptor (HBA) and oxalic acid (OX), malonic acid (MA), ethylene glycol (EG), glycerol (G), and urea (U) as hydrogen bond donors (HBD), are environmentally benign due to their natural origin and non-toxic nature. Firstly, the solubility of FCZ was experimentally determined in the presence of each DES within a temperature range of 298 K to 313 K. The obtained solubility data were subsequently correlated using thermodynamic models such as Wilson and electrolyte non-random two-liquid (e-NRTL) to provide a deeper understanding of the solute–solvent interactions. Furthermore, thermodynamic parameters of dissolution, including enthalpy, entropy, and Gibbs free energy were calculated using the Van't Hoff and Gibbs equations to elucidate the driving forces behind the dissolution process. Secondly, a fluorescence spectroscopic method was employed to investigate the molecular interactions between FCZ and the DESs. This technique provided insights into the quenching mechanism, association constants, and the number of binding sites involved in the drug–DES complexes.^15,18–20^ Finally, density functional theory (DFT) calculations, utilizing the Dmol3 and COSMO modules, were performed to further explore the interactions at the molecular level. These calculations provided valuable information on molecular descriptors such as the surface area and volume of the solute cavity, solvation energy, and electronic properties (HOMO, LUMO, and their respective energy levels), offering a more comprehensive understanding of the drug–DES interactions.^7,21^ This multi-pronged approach, combining experimental solubility measurements, thermodynamic modeling, fluorescence spectroscopy, and DFT calculations, provides a comprehensive and insightful investigation into the behavior of fluconazole in choline chloride-based DESs.

## 2. Chemicals and methods

### 2.1 Chemicals

Analytical-grade choline chloride, oxalic acid, malonic acid, ethylene glycol, glycerol, and urea were procured from Merck (Darmstadt, Germany). Fluconazole was obtained from Zahravi Pharmaceutical Company (Tabriz, Iran). HPLC-grade ethanol was supplied by Sigma-Aldrich Co., Ltd (St. Louis, MO, USA). Double-distilled deionized water was used throughout the experimental procedures. All chemicals were used as received without further purification. Table 1 provides a summary of the chemicals employed in this study.

**Table 1:** Descriptions of the used chemicals

Chemical name	Provenance	Molar mass (g mol^−1^)	CAS. no	Mass fraction (purity)	Structure
Fluconazole	Zahravi	306.271	—	>0.98	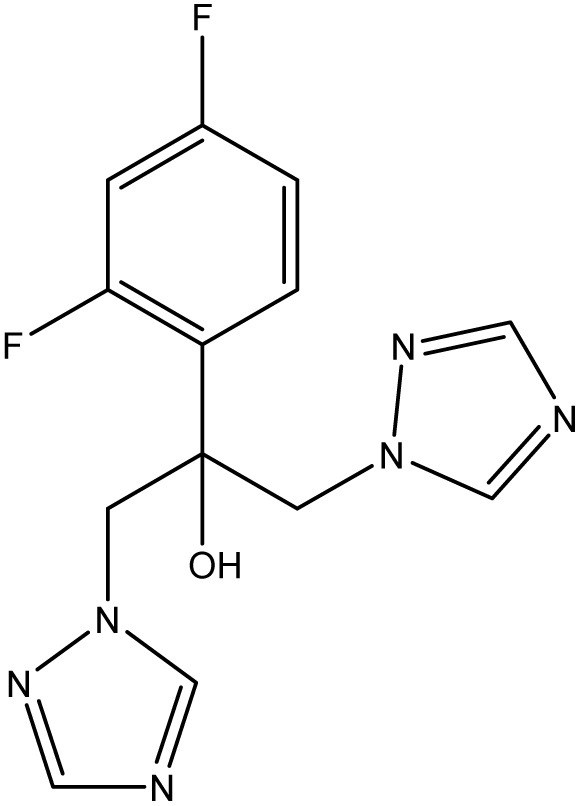
Choline choloride	Merck	139.623	67-48-1	>0.99	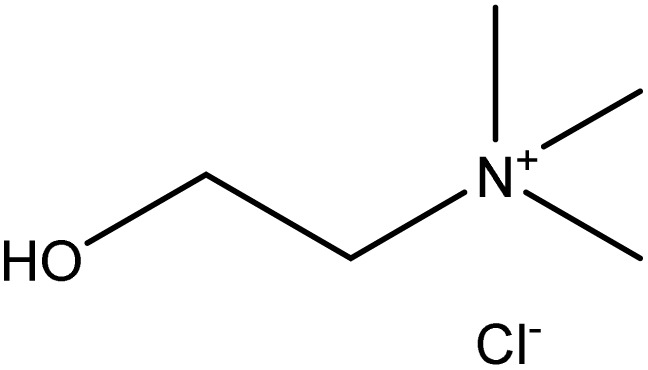
Urea	Merck	60.060	57-13-6	>0.98	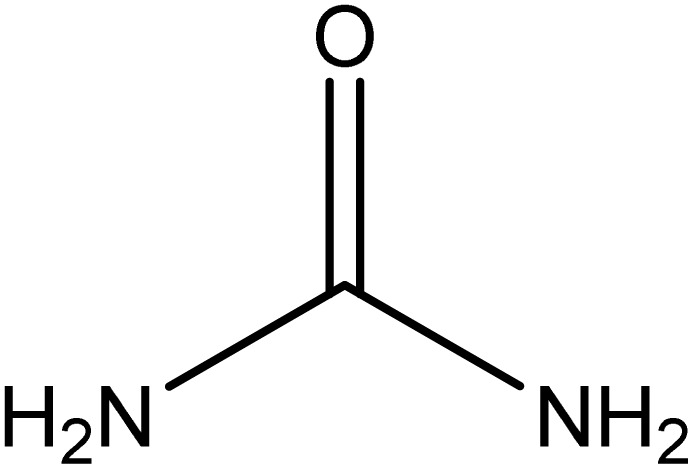
Ethylene glycol	Merck	62.070	107-21-1	>0.99	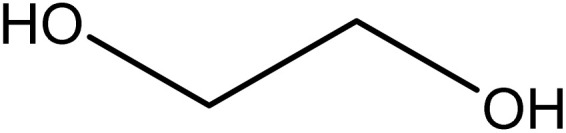
Glycerol	Merck	92.094	56-81-5	>0.99	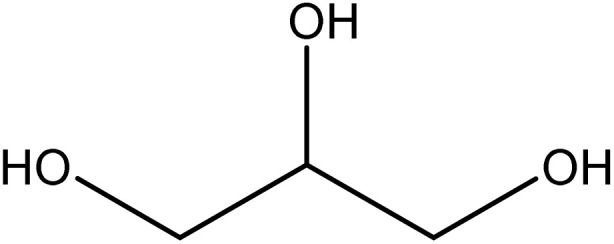
Oxalic acid	Merck	90.030	144-62-7	>0.99	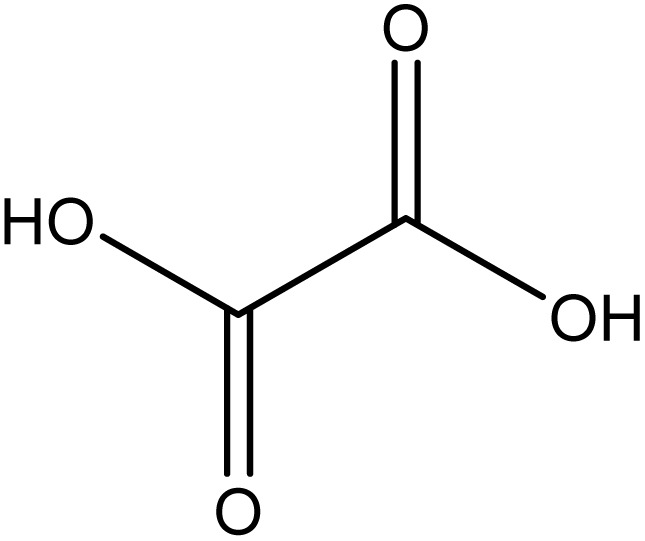
Malonic acid	Merck	104.062	141-82-2	>0.99	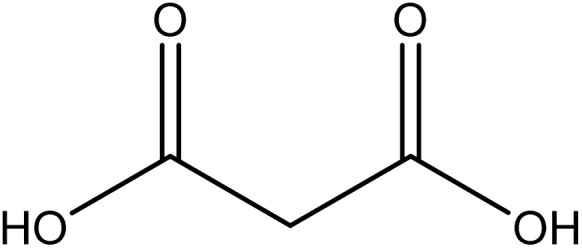

### 2.2 Preparation of deep eutectic solvents

In this study, five kinds of ChCl-based DESs (choline chloride as HBA and oxalic acid, malonic acid, ethylene glycol, glycerol and urea as HBD) with specific ratios were prepared. According to the procedure described previously, for preparing DESs, first HBA and HBD were mixed in a 50 mL round-bottom flask. The flask was then placed in a small paraffin oil-bath heated by a hot plate stirrer. The mixture was stirred for 2 h at 353 K until a colorless, homogeneous liquid formed. A thermometer (±0.1 K) was used to continually check the temperature. After processing, all samples were stored in well-sealed vials in a moisture-controlled environment. The temperature of the DES was continuously monitored with a thermometer (±0.1 K). The samples were provided in a moisture-controlled environment and then after preparation they kept in well-sealed vials. The Karl-Fischer analysis was used to measure the water content of the prepared DESs. Table 2 gives some of these solvents properties and these values compared with those reported in literature. The density and speed of sound measurements for the studied DESs were conducted using a high-precision vibrating tube digital densimeter (DSA 5000, Anton Paar, Austria), operating at a frequency of approximately 3 MHz. The densimeter was periodically calibrated using ultra-pure water and dry air as reference fluids. The instrument was equipped with a Peltier device, ensuring a stable temperature with a precision of 0.001 K. The standard uncertainties associated with density and sound speed measurements were 0.06 × 10^−3^ g cm^−3^ and 1.0 m s^−1^, respectively. The refractive index of the solutions was measured using a high-precision digital refractometer (Mettler Toledo), with an accuracy of ±0.0002 units. The refractometer was calibrated using standard calibration fluids to ensure measurement reliability.

**Table 2:** Common properties of DESs used in this work at 298.15 K^a^

DES	HBA-HBD (molar ratio)	Melting point (K)	Water content	*M*_DES_ (g mol^−1^)	10^−3^*ρ* (kg m^−3^)		*u* (m s^−1^)	*n*_D_	
					Exp	Lit		Exp	Lit
ChCl/U	1 : 2	285.15 (ref. 22)	0.06%	259.74	1.193926	1.1979 (ref. 22)	2062.27	1.5041	1.5044 (ref. 22)
ChCl/MA	1 : 1	283.15 (ref. 23)	0.07%	243.68	1.251470	1.2500 (ref. 23)	1962.69	1.4887	1.4871 (ref. 23)
ChCl/OA	1 : 1	307.15 (ref. 22)	0.32%	229.65	1.210926	1.2200 (ref. 22)	1925.00	1.4809	1.4868 (ref. 22)
ChCl/G	1 : 2	233.15 (ref. 23)	0.09%	323.80	1.176963	1.1800 (ref. 23)	2012.59	1.4865	1.4867 (ref. 23)
ChCl/EG	1 : 2	207.15 (ref. 24)	0.05%	263.76	1.116072	1.1200 (ref. 24)	1911.04	1.4685	1.4682 (ref. 24)

a The combined standard uncertainty for density is approximately *u*_c_(*ρ*) = 0.07 kg m^−3^ with a level of confidence of 0.68. Similarly, the combined standard uncertainty for the speed of sound is *u*_c_(*u*) = 0.9 m s^−1^ with level of confidence of 0.68, *u*(*n*_D_) = 0.0002, *u*(*T*) = 0.1 K.

### 2.3 Solubility determination

**Fig. 1:**
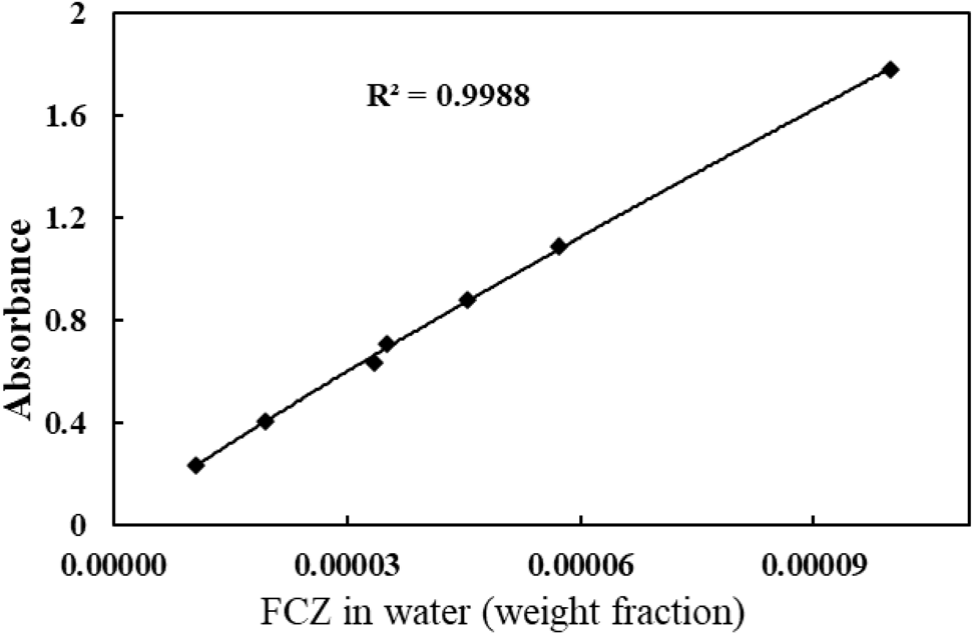
The calibration curve of the FCZ.

The solubility of fluconazole (FCZ) in various choline chloride (ChCl)-based deep eutectic solvents (DESs) was determined using the saturation shake-flask method, which is widely recognized for its accuracy and reliability. Experiments were conducted over a temperature range of (298.15 to 313.15) K, with temperature control maintained using a thermostatically regulated water bath (ED, Julabo Co., Germany) with a precision of ±0.01 K. For each measurement, a precisely weighed mass of the solvent mixture (DES + water) was introduced into sealed vials, followed by the addition of an excess amount of FCZ to ensure saturation. The Behdad shaker was employed for vigorous agitation at a temperature above the target experimental temperature for 24 hours, ensuring thorough mixing. The solid–liquid mixtures were then placed in the water bath and allowed to equilibrate undisturbed for three days to achieve saturation. After equilibration, the samples were left undisturbed for an additional seven hours to allow for phase separation. The saturated solutions were subsequently filtered using 0.22 μm PTFE filters (Whatman) or 0.44 μm hydrophilic Durapore® membrane filters (Millipore) to remove undissolved FCZ. Preliminary adsorption studies confirmed negligible drug retention on the filter media. The resulting clear solutions were diluted using a water–ethanol mixture (2 : 8 v/v) prior to spectroscopic analysis. The concentration of FCZ in the diluted solutions was determined using a T80 UV-vis spectrometer (Japan) at a wavelength of 265 nm. A calibration curve (Fig. 1) was generated using water as the background solvent, demonstrating a strong correlation coefficient of 0.9986, ensuring high accuracy.

**Fig. 2:**
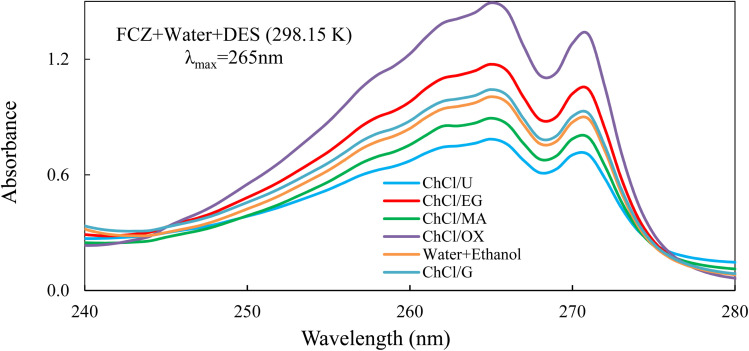
UV-vis spectra of samples (FCZ + water) and (FCZ + water + DES).

The *λ*_max_ of FCZ was observed at about 265 nm, and no significant shift in this value was detected in the presence of the DESs. Furthermore, UV-vis spectral analysis confirmed the absence of any spectral interference from the DESs on the FCZ absorbance, as depicted in Fig. 2.

Each solubility measurement was performed at least three times, and the final solubility values were reported as the mean of triplicate experiments.

The mole fraction solubility (*x*_1_) of FCZ at a certain temperature is calculated as follows:1
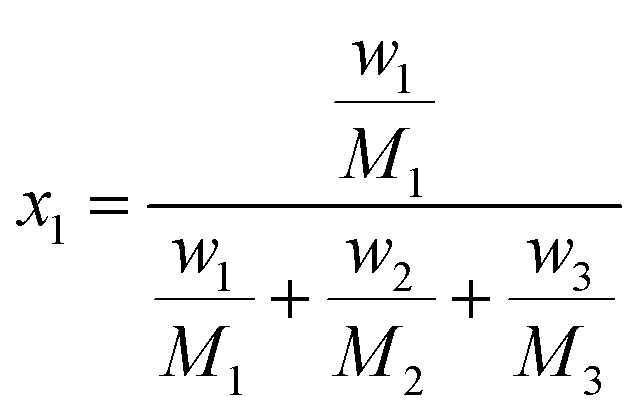
where *M*_1_, *M*_2_, *M*_3_ are the molar mass of FCZ, water, and DES respectively. Also, *w*_1_, *w*_2_, *w*_3_ represent the weight fractions of the FCZ, water, and DESs in the saturated solutions, respectively.

### 2.4 Preparation of fluconazole and DESs stock solutions

The fluconazole (FCZ) aqueous solution was prepared at a concentration of 67 μM. Concurrently, stock solutions of each deep eutectic solvent (DES) were prepared at a concentration of 187 mM in water.

### 2.5 Fluorescence spectroscopic measurements

The FCZ stock solution to obtain mixtures of a series of concentrations was mixed with DES stock solution. The final concentration of FCZ was kept constant at 20 μM, whereas the series of each DES concentrations in the FCZ-DES mixtures are (0, 37, 55, 82, 110, 146, 182 μM), (0, 36, 58, 85, 110, 150, 183 μM), (0, 35, 56, 90, 112, 153 μM), (0, 35, 56, 90, 112, 153 μM), and (0, 35, 56, 90, 112, 153 μM) for ChCl/OX, ChCl/MA, ChCl/EG, ChCl/G, and ChCl/U, respectively.

For fluorescence measurements, each FCZ–DES mixture was placed in a water bath at 298.15 K for 4 hours. The fluorescence spectra of the drug in FCZ–DES mixtures were recorded by using a fluorescence spectrophotometer (F-4500, Hitachi, Tokyo, Japan), at an excitation wavelength of 260 nm and an emission wavelength range of 265–325 nm. The voltage of the photomultiplier tube was set at 700 V, also, the emission and excitation slit widths were set at 5 nm in turn.

## 3. Results and discussion

### 3.1 Solubility studies results

**Fig. 3:**
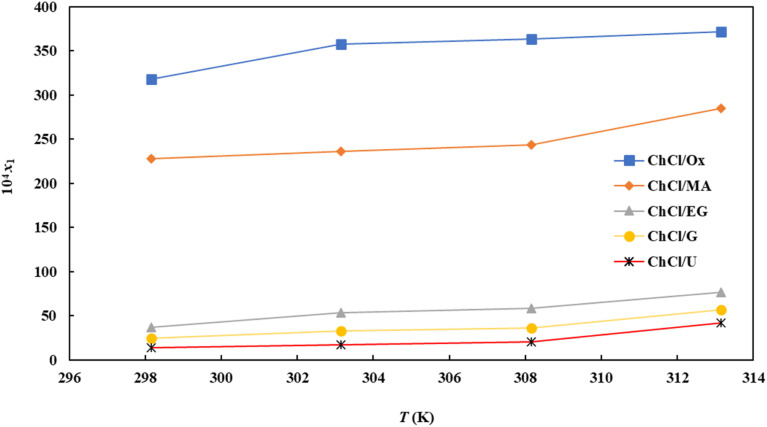
The solubility mole fraction of FCZ *versus* temperature in aqueous DESs solutions (*w*_3_ = 0.9).

The solubility of FCZ in aqueous DES mixtures at various temperatures, as expressed in mole fraction has been depicted in Fig. 3.

It is important to emphasize that the findings presented in Table 3 and Fig. 3 consistently demonstrate a similar trend in solubility.

**Table 3:** Experimental (*x*^exp^_1_)^a^ and calculated (*x*^cal^_1_) solubility of FCZ in the aqueous DES solutions at different temperatures (*T*)^b^ and weight fractions of DESs (*w*_3_)^c^ from e-NRTL and Wilson models

*T* (K)	e-NRTL model			Wilson model	
	10^4^*x*_1_^exp^	10^4^*x*_1_^cal^	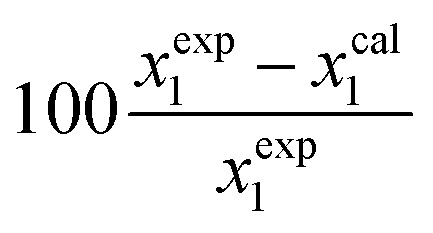	10^4^*x*_1_^cal^	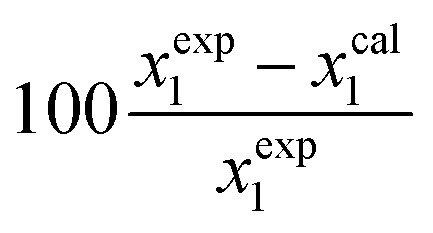
FCZ (1) + water (2) + ChCl/OX (3)					
*w*_3_ = 0.0000					
298.15	0.789	0.790	−0.03	0.790	0.02
303.15	0.831	0.817	1.71	0.830	0.06
308.15	0.879	0.890	−1.26	0.878	0.01
313.15	0.918	0.916	0.25	0.918	0.03
*w*_3_ = 0.1000					
298.15	21.602	21.055	2.53	21.585	0.08
303.15	22.224	21.899	1.46	22.203	0.09
308.15	26.400	25.556	3.2	26.336	0.05
313.15	26.630	25.935	1.76	26.616	0.25
*w*_3_ = 0.3000					
298.15	41.232	37.964	7.93	41.257	−0.06
303.15	41.452	39.167	5.51	41.446	0.02
308.15	41.740	40.583	2.77	41.779	−0.09
313.15	46.541	43.109	7.37	46.583	−0.09
*w*_3_ = 0.5000					
298.15	66.445	61.954	6.76	66.444	0.01
303.15	70.060	65.205	6.93	70.009	0.07
308.15	74.532	68.141	8.58	74.595	−0.04
313.15	76.999	73.365	4.72	77.030	−0.08
298.15	0.789	0.790	−0.03	0.790	0.02
303.15	0.831	0.817	1.71	0.830	0.06
308.15	0.879	0.890	−1.26	0.878	0.01
313.15	0.918	0.916	0.25	0.918	0.03
*w*_3_ = 0.7000					
298.15	128.711	114.569	10.99	128.275	0.34
303.15	145.157	133.082	8.32	144.737	0.29
308.15	146.318	132.803	9.24	144.950	0.93
313.15	152.131	139.432	8.35	152.000	0.09
*w*_3_ = 0.9000					
298.15	317.720	294.101	7.43	319.343	−0.51
303.15	358.173	332.669	7.12	366.012	−2.19
308.15	363.650	340.019	6.5	359.875	1.04
313.15	371.778	346.617	6.77	371.013	0.21
*w*_3_ = 1.0000					
298.15	659.940	671.264	−1.72	657.788	0.33
303.15	671.013	665.110	0.88	667.714	0.49
308.15	708.304	708.189	0.02	712.977	−0.66
313.15	747.646	737.608	1.34	748.421	−0.1

FCZ (1) + water (2) + ChCl/MA (3)					
*w*_3_ = 0.0000					
298.15	0.789	0.790	−0.03	0.790	0.02
303.15	0.831	0.817	1.71	0.830	0.06
308.15	0.879	0.890	−1.26	0.878	0.01
313.15	0.918	0.916	0.25	0.918	0.03
*w*_3_ = 0.1000					
298.15	12.43	12.310	0.99	12.378	0.44
303.15	12.981	12.522	3.54	12.900	0.63
308.15	13.294	12.769	3.95	13.319	−0.19
313.15	14.055	13.521	3.81	14.063	−0.05
*w*_3_ = 0.3000					
298.15	28.720	27.355	4.75	28.638	0.28
303.15	30.716	29.561	3.76	30.500	0.70
308.15	30.681	29.496	3.86	30.596	0.28
313.15	31.760	30.493	3.99	31.816	−0.18
*w*_3_ = 0.5000					
298.15	63.544	57.869	8.93	63.320	−0.35
303.15	74.686	65.893	11.77	74.176	0.68
308.15	75.014	65.988	12.03	74.538	0.63
313.15	75.576	67.359	10.87	75.643	−0.09
*w*_3_ = 0.7000					
298.15	110.105	99.225	9.88	110.004	0.09
303.15	114.613	104.637	8.7	114.487	0.11
308.15	118.059	108.766	7.26	108.035	0.08
313.15	113.714	109.354	9.99	113.592	0.02
*w*_3_ = 0.9000					
298.15	228.379	217.586	4.73	228.374	0.01
303.15	236.138	217.586	3.56	235.945	0.08
308.15	243.371	228.234	6.22	242.865	−0.02
313.15	285.384	265.559	6.95	285.447	0.21
*w*_3_ = 1.0000					
298.15	387.420	393.574	−1.59	387.530	−0.03
303.15	411.048	431.082	−4.87	410.880	0.04
308.15	443.621	436.380	1.63	443.718	−0.02
313.15	474.950	462.928	2.53	475.029	−0.02

FCZ (1) + water (2) + ChCl/EG (3)					
*w*_3_ = 0.0000					
298.15	0.789	0.790	−0.03	0.790	0.02
303.15	0.831	0.817	1.71	0.830	0.06
308.15	0.879	0.890	−1.26	0.878	0.01
313.15	0.918	0.916	0.25	0.918	0.03
*w*_3_=0.01000					
298.15	5.076	4.267	15.93	5.099	−0.47
303.15	5.510	4.757	13.64	5.521	−0.23
308.15	7.250	6.438	11.20	7.254	−0.02
313.15	7.770	6.539	15.84	7.770	−0.01
*w*_3_ = 0.3000					
298.15	6.817	6.817	2.31	6.820	−0.04
303.15	7.281	6.748	7.33	6.748	0.30
308.15	8.027	7.251	9.67	8.009	0.22
313.15	8.407	7.605	9.54	8.407	0.04
*w*_3_ = 0.5000					
298.15	10.677	10.206	4.41	10.612	0.61
303.15	10.826	10.347	4.42	10.767	0.55
308.15	11.445	11.216	2.07	11.440	0.11
313.15	15.445	15.426	5.33	15.426	0.19
*w*_3_ = 0.7000					
298.15	10.879	11.367	−4.49	10.917	−0.35
303.15	15.830	15.349	3.03	15.821	0.05
308.15	22.789	22.422	1.60	22.780	0.03
313.15	29.943	29.157	2.62	29.917	0.08
*w*_3_ = 0.9000					
298.15	36.865	36.249	1.67	36.855	0.03
303.15	53.673	52.616	1.97	53.550	0.23
308.15	58.573	61.216	−4.51	58.580	−0.01
313.15	76.906	76.561	0.45	76.832	0.10
*w*_3_ = 1.0000					
298.15	207.104	212.274	−2.50	207.240	−0.07
303.15	216.172	213.080	1.40	216.071	0.01
308.15	221.399	231.249	−4.50	221.261	−0.01
313.15	226.451	227.754	−0.58	226.480	0.02

FCZ (1) + water (2) + ChCl/G (3)					
*w*_3_ = 0.0000					
298.15	0.789	0.790	298.15	0.789	0.790
303.15	0.831	0.817	303.15	0.831	0.817
308.15	0.879	0.890	308.15	0.879	0.890
313.15	0.918	0.916	313.15	0.918	0.916
*w*_3_ = 0.1000					
298.15	3.395	3.230	4.84	3.401	−0.18
303.15	5.440	5.333	1.95	5.443	−0.07
308.15	6.372	6.228	2.26	6.363	0.14
313.15	7.452	7.273	2.41	7.461	−0.12
*w*_3_ = 0.3000					
298.15	3.855	3.857	−0.04	3.842	0.35
303.15	6.194	6.558	−5.89	6.163	0.50
308.15	7.820	8.089	−3.45	7.811	0.11
313.15	8.102	8.156	−0.67	8.134	0.40
*w*_3_ = 0.5000					
298.15	4.816	4.373	9.20	4.801	0.22
303.15	9.469	8.831	6.73	9.416	0.55
308.15	10.652	9.972	6.39	10.595	0.54
313.15	13.498	11.862	12.12	13.457	0.31
*w*_3_ = 0.7000					
298.15	7.923	8.970	−13.22	7.976	−0.68
303.15	15.801	17.504	−10.78	15.848	−0.29
308.15	16.902	18.616	−10.14	16.875	0.16
313.15	21.846	23.666	−8.33	22.002	−0.72
*w*_3_ = 0.9000					
298.15	24.403	23.770	2.59	24.407	−0.02
303.15	32.679	32.724	−0.14	32.676	0.01
308.15	36.088	35.319	2.13	36.085	0.01
313.15	56.526	53.597	5.18	56.528	0.00
*w*_3_ = 1.0000					
298.15	96.810	97.140	−0.34	96.811	0.00
303.15	126.534	126.179	0.28	126.502	0.03
308.15	158.089	158.119	−0.02	158.069	0.01
313.15	172.782	174.561	−1.03	172.687	0.05

FCZ (1) + water (2) + ChCl/U (3)					
*w*_3_ = 0.0000					
298.15	0.789	0.790	−0.03	0.790	0.02
303.15	0.831	0.817	1.71	0.830	0.06
308.15	0.879	0.890	−1.26	0.878	0.01
313.15	0.918	0.916	0.25	0.918	0.03
*w*_3_ = 0.1000 (ref. 1)					
298.15	3.224	3.218	0.20	3.226	−0.05
303.15	4.795	4.536	5.40	4.827	−0.66
308.15	6.156	5.977	2.90	6.156	−0.01
313.15	6.617	6.286	5.01	6.615	5.01
*w*_3_ = 0.3000					
298.15	3.806	3.754	1.33	3.783	−0.05
303.15	6.039	5.989	0.83	6.036	−0.66
308.15	7.716	7.735	−0.25	7.732	−0.21
313.15	7.878	8.317	−5.57	7.882	0.04
*w*_3_ = 0.5000					
298.15	4.677	4.727	−1.05	4.650	0.57
303.15	7.298	8.385	−14.89	7.285	0.18
308.15	10.110	10.505	−3.91	10.192	−0.81
313.15	12.364	12.144	1.78	12.362	−0.05
*w*_3_ = 0.7000					
298.15	7.049	6.898	2.14	6.991	0.82
303.15	14.549	12.309	15.4	14.347	1.39
308.15	15.933	14.703	−0.8	15.922	0.07
313.15	21.736	20.532	1.06	21.684	0.04
*w*_3_ = 0.9000					
298.15	14.037	13.877	1.14	13.395	0.74
303.15	17.237	17.781	−3.15	17.219	0.10
308.15	20.250	20.411	−0.80	20.480	−1.14
313.15	41.619	41.179	1.06	41.603	0.04
*w*_3_ = 1.000					
298.15	23.799	23.654	0.61	23.662	0.57
303.15	26.648	26.637	0.04	26.502	0.55
308.15	34.254	34.932	−1.98	34.553	−0.87
313.15	57.183	57.398	−0.37	57.129	0.10

a Standard uncertainty of experimental solubility is *u*(*x*^exp^_1_) = 0.07.

b Standard uncertainty of temperature is *u*(*T*) = 0.01 K.

c Standard uncertainty of mass fraction is *u*(*w*_3_) = 0.0005.

Specifically, as the temperature and concentration of DESs increase, the solubility of FCZ also increases. These results indicate that the solubility of FCZ is higher in aqueous DESs compared to pure water.^20,25^ This can be attributed to the interactions between certain components of FCZ and DESs, as depicted in Fig. 3.

The ordering of FCZ solubility in the presence of DESs is as follows:ChCl/OX > ChCl/MA > ChCl/EG > ChCl/G > ChCl/U

The observed solubility enhancement of fluconazole (FCZ) in deep eutectic solvents (DESs) can be attributed to a complex interplay of intermolecular forces. Solubilization of hydrophobic drugs in a solvent is inherently dependent on the strength and nature of these interactions. In the case of FCZ and DESs, several key forces are likely contributing factors. Firstly, hydrogen bonding plays a significant role. Both FCZ and DESs possess functional groups capable of participating in hydrogen bond formation, such as hydroxyl (–OH) and amino (–NH) groups. These interactions facilitate the formation of solute–solvent complexes, enhancing the solubility of FCZ within the DES matrix. Secondly, van der Waals forces, including dipole–dipole interactions, contribute to the overall solvation process. These weak, attractive forces arise from temporary fluctuations in electron distribution within the molecules, leading to transient dipoles that interact with each other. Thirdly, the presence of ionic species within DESs introduces ion–dipole interactions. These interactions arise from the attraction between the charged ions in the DES and the polar regions of the FCZ molecule. The combined effect of these intermolecular forces results in a significant enhancement of FCZ solubility in DESs compared to aqueous solutions. In aqueous systems, solvation primarily relies on hydrogen bonding and dipole–dipole interactions between FCZ and water molecules. However, the introduction of DESs introduces additional, stronger interactions, notably ion–dipole interactions, leading to a more favorable solvation environment for the drug.^1,4,18,26–31^

### 3.2 Correlation procedure

The thermodynamic models hold paramount importance within the pharmaceutical industry, serving as crucial tools for assessing drug solubility and guiding the selection of appropriate solvents for formulation development. Accurate prediction of solubility across various solvent systems is achieved through the resolution of equilibrium thermodynamic equations. Modern theoretical frameworks for solubility prediction often incorporate local composition theories. These theories elegantly account for the intricate nuances of molecular interactions, specifically addressing the short-range order and non-random molecular orientations that arise from variations in molecular size and intermolecular forces within the solution.^32,33^ These equations utilize the excess molar Gibbs energy (*G*^ex^) to capture such effects. To determine the solute's solubility in a solution at specific temperatures, the solute's activity in the saturated solution is equated to its activity in the pure solid state. This equilibrium condition can be described using a solid–liquid equilibrium (SLE) framework, yielding the following equation:2
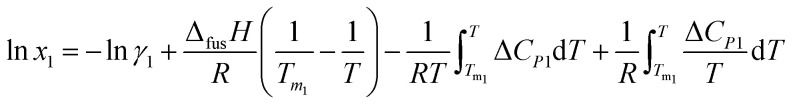


In the equation, the symbols have the following meanings: *R* represents the gas constant, *T* represents the experimental temperature, and *T*_m_1__ represents the melting temperature. The variables Δ_fus_*H*, *γ*_1_, and Δ*C*_P1_ represent the enthalpy of fusion, activity coefficient, and difference in molar heat capacity between the melted and solid states of FCZ, respectively. Finally, by employing reasonable approximations, the simplified equation is obtained as follows:^15^3
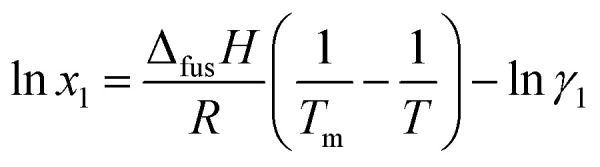


To obtain the experimental solubility data of FCZ, it is necessary to determine the melting temperature (*T*_m_), activity coefficient (*γ*_1_), and enthalpy of fusion (Δ_fus_*H*). In this study, the correlation of these parameters has facilitated the analysis. The molar excess Gibbs energy (*G*^ex^) is expressed as the sum of two contributions, allowing for the generalization of the e-NRTL and Wilson models to encompass multicomponent systems that include electrolytes dissolved in aqueous solutions:^15,20^4
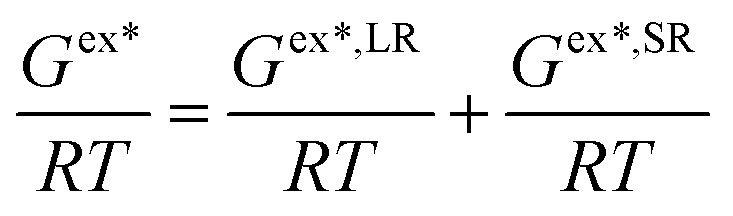


Superscript * denotes asymmetric convention, SR represents short-range interaction, and LR indicates long-range interaction. The Pitzer-Debye Hückel extended model (*G*^ex*^) developed by Pitzer (1980)^34^ offers a suitable approach for capturing long-range interaction effects. Additionally, for short-range interactions, the e-NRTL and Wilson models have been utilized.

#### 3.2.1 Wilson model

In the Wilson model, the activity coefficients, which depend primarily on composition and temperature, are defined by the following equations:^35^5
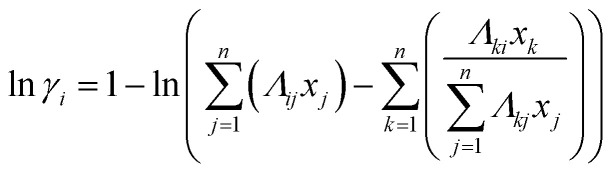


In the equations, the variable *λ* represents the binary interaction parameter determined by the characteristic energy (*∧*_*ij*_) and the molar volumes of the solute and solvent (*ν*). The calculation of this parameter can be performed using eqn (6):^20^6
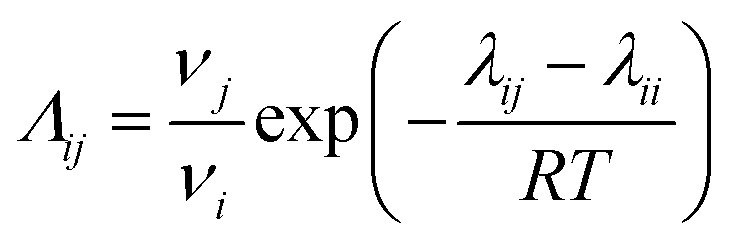


#### 3.2.2 e-NRTL model

The application of the local composition concept in the context of the electrolyte nonrandom two-liquid (e-NRTL) equation has been extensively discussed in previous studies.^4,15,18,26–31^ To calculate the activity coefficient for each species, a combination of the Pitzer-Debye-Hückel contribution and the NRTL contribution is employed. This approach allows for the evaluation of (*γ*_1_) using eqn (7) and (8):^36,37^7
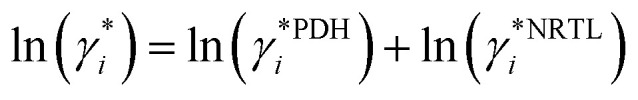
8
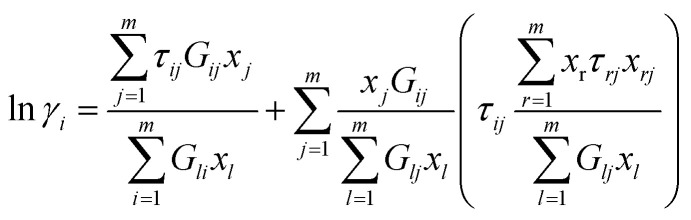
9
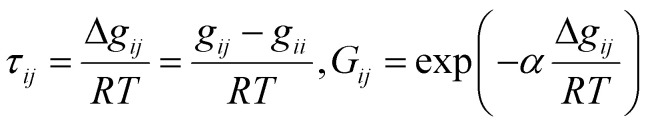
where *G*_*ij*_ were identified as *G*_*ij*_ = exp(−*α*_*ij*_*τ*_*ij*_), *τ*_*ii*_ = *τ*_*jj*_ = 0 and *α*_*ij*_ = *α*_*ji*_ (non-randomness parameter). Also, eqn (9) has also been used to calculate the binary interaction parameter (*τ*_*ij*_)

The *g*_*ij*_ is an energy parameter characteristic of the *i*–*j* interactions.

Finally, the interaction parameters of the models were determined by minimizing the objective function eqn (10).10
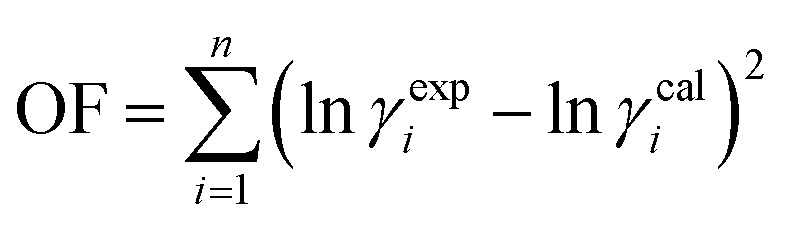


The activity coefficients of the obtained states are denoted as ln *γ*^cal^_*i*_ and *γ*^exp^_*i*_. To assess the difference between calculated (*x*^cal^_*i*_) and experimental (*x*^exp^_*i*_) solubility data, the relative deviation percent (ARD percent) can be employed. The ARD percent is determined using eqn (11), which is applicable to the aforementioned thermodynamic models. In this equation, *N* represents the number of experimental points:11
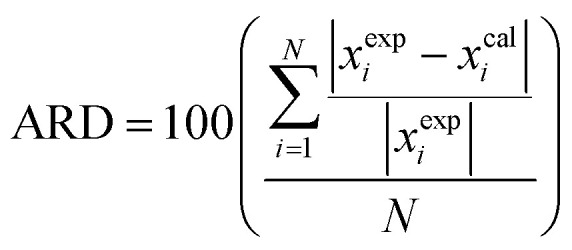


The solubility data was correlated using the Wilson and e-NRTL models, and the resulting outcomes and relevant parameters have been compiled in Table 3. Additionally, Table 4 presents the values of the ARD percentage, which indicates a low ARD%, signifying that all models demonstrate satisfactory accuracy in predicting the mole fraction solubility of FCZ in these systems.^38–40^

**Table 4:** The calculated average relative deviation percent (ARD%) for the solubility of the FCZ in the aqueous DESs solutions at the temperature ranges *T* (K) = 298.15 to 313.15 and pressure (*p* = 866 hPa) from different models

ARD%		
*T* (K)	e-NRTL	Wilson
FCZ (1) + water (2) + ChCl/EG (3)		
298.15	4.48	0.24
303.15	4.78	0.21
308.15	4.97	0.06
313.15	4.94	0.07
Average	4.79	0.12

FCZ (1) + water (2) + ChCl/G (3)		
298.15	8.51	0.30
303.15	6.62	0.25
308.15	7.72	0.18
313.15	6.29	0.25
Average	7.28	0.24

FCZ (1) + water (2) + ChCl/U (3)		
298.15	1.08	0.48
303.15	6.62	0.42
308.15	2.92	0.44
313.15	3.21	0.07
Average	3.46	0.35

FCZ (1) + water (2) + ChCl/MA (3)		
298.15	5.80	0.32
303.15	5.89	0.72
308.15	5.93	0.64
313.15	5.85	0.13
Average	5.80	0.45

FCZ (1) + water (2) + ChCl/OX (3)		
298.15	5.57	0.04
303.15	5.18	0.07
308.15	4.94	0.10
313.15	5.55	0.05
Average	5.32	0.07

The results indicate a strong agreement between the models and the experimental data, highlighting their excellent precision. The correlation performance for aqueous solutions containing ARD% is as follows: Wilson (0.20) > e-NRTL (5.34).

### 3.3 The apparent dissolution thermodynamic properties

The thermodynamic properties of the dissolution process were determined by evaluating apparent thermodynamic functions using the Gibbs and van't Hoff equations. These calculations were performed at the mean harmonic temperature 
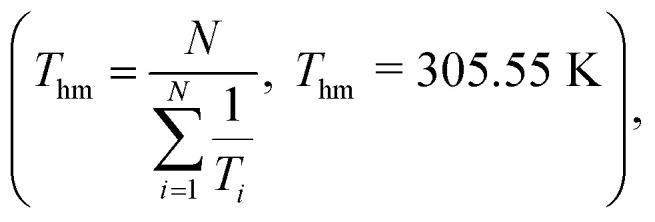
 which was obtained from the temperature range of 298.15 K to 313.15 K.^41–44^ The standard molar enthalpy of dissolution (
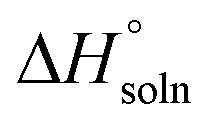
) for FCZ was determined using eqn (12):12
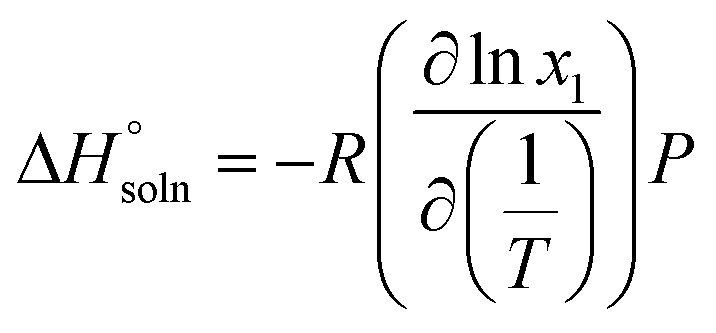


In the equation, *x*_1_ denotes the mole fraction of FCZ, *R* is the universal gas constant with a value of 8.314 J K^−1^ mol^−1^, and *T* represents the absolute temperature. 
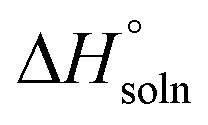
 was also computed by graphing ln *x*_1_*versus*
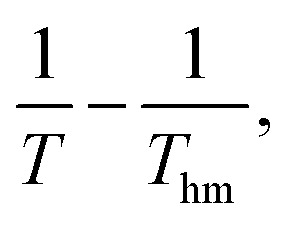
 which known as the Van't Hoff plot:^15^13
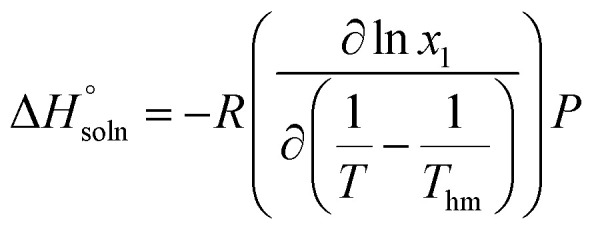
14
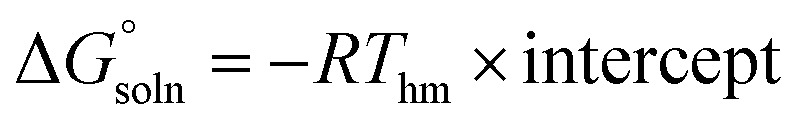


By utilizing the slope and intercept of eqn (13) and (14), the values of 
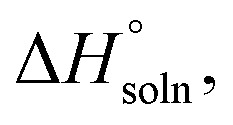
 and 
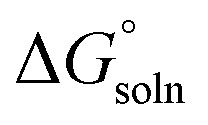
 have been determined. Moreover, the standard molar entropy of dissolution, 
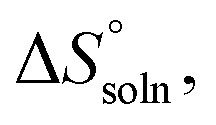
 was obtained by the following equation:15
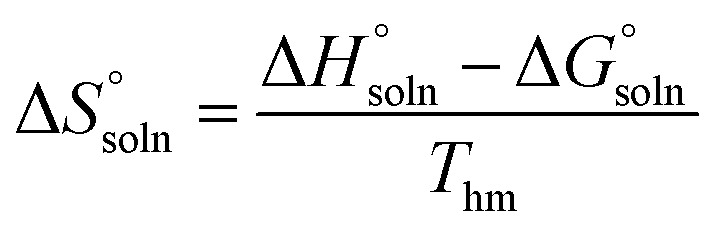


Eventually, in the FCZ dissolving process, eqn (16) and (17) have been applied to compare the relative contributions to the standard molar Gibbs free energy by enthalpy and entropy, as evidenced by the *ξ*_H_ and *ξ*_TS_ values:^15,20^16
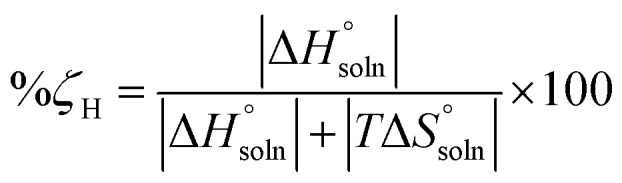
17
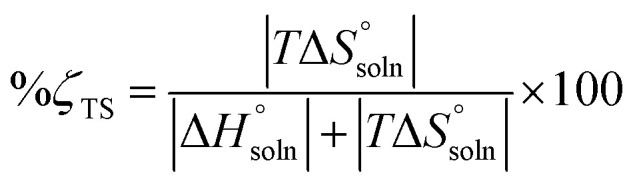


In order to assess the thermodynamic properties of the dissolution process, the plots of (ln *x*_1_) *versus*
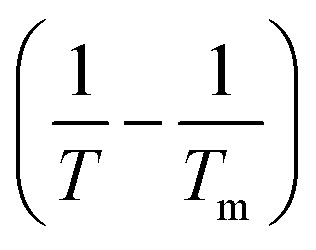
 for FCZ in aqueous DESs solutions was graphically represented. Additionally, the collected data for 
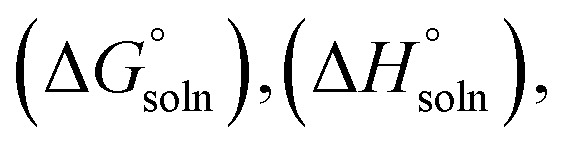
 and 
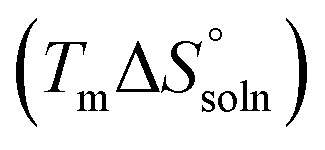
 are presented in Table 5.

**Table 5:** Thermodynamic functions for dissolution process at different weight fractions of PILs (*w*_3_) at mean harmonic temperature (*T*_hm_ = 305.55 K)

*w*_3_	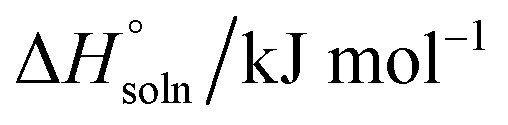	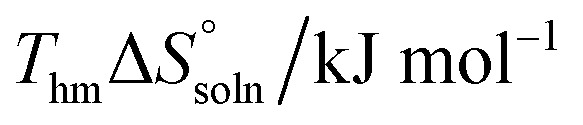	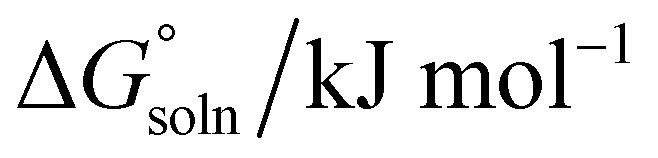	*ξ*_H_	*ξ*_TS_
FCZ (1) + water (2) + ChCl/EG (3)					
0.0000	7.87	−15.93	23.80	33.08	66.92
0.1000	24.09	5.37	18.72	81.76	18.24
0.3000	11.28	−6.96	18.24	61.86	29.46
0.5000	17.95	0.86	17.09	95.44	4.56
0.7000	52.86	36.87	15.98	58.91	41.09
0.9000	35.65	22.42	13.23	61.39	38.60
1.0000	4.54	−5.18	11.23	46.68	12.70

FCZ (1) + water (2) + ChCl/GLY (3)					
0.0000	7.87	−15.93	23.80	33.08	66.92
0.1000	39.23	20.14	19.09	66.08	33.92
0.3000	38.43	19.68	18.75	66.13	34.66
0.5000	50.05	32.23	17.82	60.83	39.17
0.7000	48.50	31.93	16.58	60.30	39.69
0.9000	30.48	17.07	13.41	64.10	35.90
1.0000	23.15	10.45	12.70	68.89	25.54

FCZ (1) + water (2) + ChCl/U (3)					
0.0000	7.87	−15.93	23.80	33.08	66.92
0.1000	37.52	18.21	19.30	67.32	32.68
0.3000	37.92	19.12	18.80	66.48	31.93
0.5000	50.44	32.36	18.09	60.92	39.08
0.7000	68.82	52.28	16.54	56.83	43.17
0.9000	85.28	69.21	14.68	55.20	44.80
1.0000	44.52	30.03	14.19	59.72	26.04

FCZ (1) + water (2) + ChCl/MA (3)					
0.0000	7.87	−15.93	23.80	33.08	66.92
0.1000	12.43	−2.88	16.85	81.16	18.84
0.3000	5.69	−8.17	14.72	41.08	20.81
0.5000	7.83	−4.70	12.54	62.50	37.50
0.7000	14.09	2.92	11.16	82.81	17.19
0.9000	10.78	1.38	9.46	88.63	11.37
1.0000	10.66	2.66	8.16	80.04	19.79

FCZ (1) + water (2) + ChCl/OX (3)					
0.0000	7.87	−15.93	23.80	33.08	66.92
0.1000	6.08	−10.77	15.31	36.07	63.93
0.3000	4.72	−10.00	13.86	32.07	73.17
0.5000	8.22	−4.31	12.16	65.60	34.39
0.7000	7.95	−2.84	10.79	73.66	26.34
0.9000	7.60	−0.90	8.79	89.44	10.56
1.0000	3.01	−3.84	6.85	43.91	33.57

It is noteworthy that all systems exhibited positive values for the standard molar Gibbs energy and dissolution enthalpy in the FCZ dissolution process within aqueous DES solutions, indicating that the dissolution processes are consistently endothermic. The 
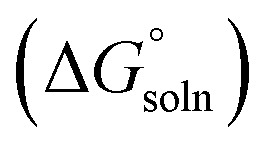
 values decline as the weight fraction of DES increases, illustrating that the solubility of DES in these types of solvents increases as the 
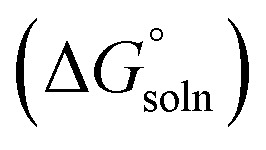
 values decrease. On the other hand, 
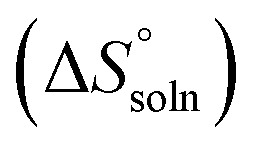
 is positive in all investigated in this study solutions, and its values as 
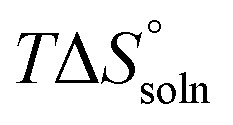
 are lower than those of 
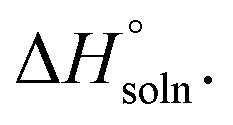
Table 6 depicts the computed (*ξ*_H_) and (*ξ*_TS_) values. Based on the mentioned data, the enthalpy is the major contribution to the standard molar Gibbs energy of the dissolution process of DES.^31,45,46^

The parameters of the e-NRTL model were applied to analyze the behavior of FCZ in aqueous DES solutions

**Table:** 

*T* (K)	10^−4^ Δ*g*_wd_	10^−3^ Δ*g*_dw_	10^−3^ Δ*g*_EGd_	10^−4^ Δ*g*_dEG_	Δ*g*_caw_	Δ*g*_wca_	10^−4^ Δ*g*_cad_	Δ*g*_dca_	10^−3^ Δ*g*_EGca_	10^−4^ Δg_caEG_	10^−4^ Δ*g*_EGw_	10^−3^ Δ*g*_wEG_	RMSD (σ)
FLU (1) + water (2) + ChCl/EG (3)													
298.15	4.23	−14.68	−5.25	−12.22	0.64	4.23	−2.41	7.15	0.75	−14.74	−2.80	4.97	4.477
303.15	3.13	−9.39	−4.86	−6.98	0.64	4.23	−1.47	7.16	0.73	−9.47	−2.13	4.69	4.786
308.15	4.53	−18.97	−5.65	−8.26	0.64	4.23	−2.96	7.15	0.99	−17.44	−2.31	4.93	4.971
313.15	2.75	−7.78	−4.92	−5.62	0.64	4.23	−1.39	7.16	0.71	−9.01	−1.46	4.69	4.944

**Table:** 

*T* (K)	10^−4^ Δ*g*_wd_	10^−3^ Δ*g*_dw_	10^−3^ Δ*g*_GLYd_	10^−3^ Δ*g*_dGLY_	10^−3^ Δ*g*_caw_	Δ*g*_wca_	10^−3^ Δ*g*_cad_	Δ*g*_dca_	10^−3^ Δ*g*_GLYca_	10^−4^ Δ*g*_caGLY_	10^−4^ Δ*g*_GLYw_	10^−3^ Δ*g*_wGLY_	RMSD (σ)
FLU (1) + water (2) + ChCl/GLY (3)													
298.15	3.99	−12.76	−0.33	0.31	1.60	2.243	−0.59	3.76	0.01	−1.36	−2.72	0.14	8.511
303.15	2.76	−13.28	−3.95	4.49	6.40	4.24	−9.48	7.17	4.54	−1.70	−0.12	1.86	6.627
308.15	3.82	−2.08	−5.37	0.47	0.64	4.22	−26.19	7.15	0.89	−25.16	−0.13	3.61	7.729
313.15	2.20	−9.20	−4.37	4.69	0.64	4.24	−11.15	7.17	0.44	−16.17	−0.01	1.83	6.290

**Table:** 

*T* (K)	10^−4^ Δ*g*_wd_	10^−3^ Δ*g*_dw_	10^−4^ Δ*g*_Ud_	10^−4^ Δ*g*_dU_	Δ*g*_caw_	Δ*g*_wca_	10^−3^ Δ*g*_cad_	Δ*g*_dca_	10^−4^ Δ*g*_Uca_	10^−5^ Δ*g*_caU_	10^−5^ Δ*g*_Uw_	Δ*g*_wU_	RMSD (σ)
FLU (1) + water (2) + ChCl/U (3)													
298.15	1.17	−1.35	−0.37	0.26	0.16	2.25	−0.64	3.76	−1.29	−0.29	−0.03	0.15	1.080
303.15	1.73	−6.92	−23.30	0.17	0.16	2.12	−3.53	3.60	−1.24	−7.44	−2.45	0.32	6.622
308.15	1.73	−6.91	−32.41	0.17	0.16	2.12	−36.03	3.60	−1.24	−10.13	−3.38	0.32	2.917
313.15	1.79	−6.83	−22.53	0.17	0.16	2.12	−35.36	−11.79	−1.24	−7.39	−2.39	0.32	3.219

**Table:** 

*T* (K)	10^−3^ Δ*g*_wd_	10^−4^ Δ*g*_dw_	10^−4^ Δ*g*_OAd_	Δ*g*_dOA_	Δ*g*_caw_	Δ*g*_wca_	10^−4^ Δ*g*_cad_	Δ*g*_dca_	Δ*g*_OAca_	10^−4^ Δ*g*_caOA_	10^−3^ Δ*g*_OAw_	10^−3^ Δ*g*_wOA_	RMSD (σ)
FLU (1) + water (2) + ChCl/OA (3)													
298.15	5.06	1.64	−1.06	−2.21	0.64	4.25	−1.33	7.15	351.02	−9.11	2.23	2.55	5.802
303.15	5.77	1.63	−1.10	−2.53	0.64	4.25	−1.35	7.15	350.02	−9.72	2.54	2.62	5.898
308.15	3.59	2.30	−9.48	−2.83	0.64	4.25	−1.26	7.15	361.38	−9.28	8.06	2.60	5.933
313.15	5.70	1.89	−1.05	−2.36	0.64	4.25	−1.33	7.15	350.76	−9.95	2.28	2.60	5.854

**Table:** 

*T* (K)	10^−4^ Δ*g*_wd_	10^−3^ Δ*g*_dw_	10^−3^ Δ*g*_MAd_	10^−4^ Δ*g*_dMA_	Δ*g*_caw_	Δ*g*_wca_	10^−3^ Δ*g*_cad_	Δ*g*_dca_	Δ*g*_MAca_	10^−4^ Δ*g*_caMA_	10^−3^ Δ*g*_MAw_	10^−3^ Δ*g*_wMA_	RMSD (σ)
FLU (1) + water (2) + ChCl/MA (3)													
298.15	1.23	2.55	−5.92	−1.18	0.64	4.25	−8.30	7.16	327.42	−8.454	−7.99	2.39	5.569
303.15	0.81	9.22	−8.74	−9.59	0.64	4.25	−0.12	7.15	340.63	−9.80	−15.03	2.40	5.174
308.15	4.96	1.49	−6.04	0.36	0.64	4.25	−9.27	7.15	355.70	−7.20	−4.19	2.07	4.942
313.15	1.16	6.08	−4.05	−0.43	0.64	4.25	−6.29	7.16	337.32	−7.13	−6.18	1.92	5.555

### 3.4 Results obtained from the modeling

The outcomes of the computational modeling of FCZ solubility in binary aqueous DES solutions, encompassing various physicochemical parameters, are tabulated in Tables 6 and 7.

The parameters of the Wilson model were applied to analyze the behavior of FCZ in aqueous DES solutions

**Table:** 

*T* (K)	10^5^*Λ*_dw_	*Λ*_wd_	10^4^*Λ*_dEG_	*Λ*_EGd_	*Λ*_dca_	*Λ*_cad_	*Λ*_wEG_	*Λ*_EGw_	*Λ*_wca_	10^4^*Λ*_caw_	*Λ*_EGca_	*Λ*_caEG_	10^3^ RMSD (σ)
FLU (1) + water (2) + ChCl/EG (3)													
298.15	1.25	3.92	−0.01	4.58	0.03	0.03	0.30	3.49	0.01	−1.29	0.04	−0.03	2.390
303.15	2.24	3.67	−0.01	4.28	0.03	0.04	0.47	2.30	0.01	−2.47	0.04	−0.03	2.056
308.15	2.44	3.44	−0.01	3.99	0.03	0.04	0.25	3.82	0.01	−2.77	0.04	−0.03	0.618
313.15	2.60	3.22	−0.01	3.72	0.03	0.04	0.35	2.79	0.01	−2.89	0.04	−0.03	0.665

**Table:** 

*T* (K)	10^5^*Λ*_dw_	*Λ*_wd_	10^4^*Λ*_dGLY_	*Λ*_GLYd_	*Λ*_dca_	*Λ*_cad_	*Λ*_wGLY_	*Λ*_GLYw_	*Λ*_wca_	10^4^*Λ*_caw_	*Λ*_GLYca_	*Λ*_caGLY_	10^3^ RMSD (σ)
FLU (1) + water (2) + ChCl/GLY (3)													
298.15	2.03	3.91	−0.01	4.13	0.03	0.04	1.34	0.78	0.01	−1.69	0.04	−0.03	3.033
303.15	5.97	3.57	−0.01	3.81	0.03	0.12	0.18	4.41	0.01	−2.83	0.04	−0.03	2.462
308.15	2.02	2.97	−0.01	3.18	0.03	0.42	0.16	4.65	0.01	−9.10	0.04	−0.03	1.791
313.15	1.96	2.79	−0.01	2.99	0.03	0.42	0.36	2.57	0.01	−2.87	0.04	−0.03	2.516

**Table:** 

*T* (K)	10^5^*Λ*_dw_	*Λ*_wd_	*Λ*_dU_	*Λ*_Ud_	*Λ*_dca_	*Λ*_cad_	*Λ*_wU_	*Λ*_Uw_	*Λ*_wca_	10^4^*Λ*_caw_	10^3^*Λ*_Uca_	*Λ*_caU_	10^3^ RMSD (σ)
FLU (1) + water (2) + ChCl/U (3)													
298.15	5.96	3.80	−0.01	3.95	0.03	0.12	0.19	4.83	0.01	−2.80	0.04	−0.03	4.824
303.15	4.29	3.62	−0.01	3.75	0.03	0.08	0.20	4.54	0.01	−2.18	0.04	−0.03	4.193
308.15	4.61	3.39	−0.01	3.48	0.03	0.08	0.21	4.75	0.01	−2.18	0.04	−0.03	4.454
313.15	3.91	3.19	−0.01	3.36	0.03	0.06	0.22	4.03	0.01	−2.31	0.04	−0.03	0.680

**Table:** 

*T* (K)	10^6^*Λ*_dw_	*Λ*_wd_	10^4^*Λ*_dOA_	*Λ*_OAd_	10^3^*Λ*_dca_	*Λ*_cad_	*Λ*_wOA_	*Λ*_OAw_	10^3^*Λ*_wca_	10^3^*Λ*_caw_	*Λ*_OAca_	*Λ*_caOA3_	10^3^ RMSD (σ)
FLU (1) + water (2) + ChCl/OA (3)													
298.15	−12.15	0.44	7.62	0.47	−1.57	0.03	0.04	7.80	1.00	4.75	−0.02	0.03	3.216
303.15	−6.80	0.21	7.64	0.23	−1.57	0.01	0.03	3.21	0.99	5.50	−0.02	0.03	7.122
308.15	0.01	0.08	1.91	0.02	−0.40	0.01	0.01	1.73	0.25	2.76	−0.01	0.03	6.362
313.15	0.01	−0.02	0.31	−0.22	−6.25	0.02	0.01	9.28	4.00	0.01	−0.01	0.03	1.207

**Table:** 

*T* (K)	10^6^*Λ*_dw_	*Λ*_wd_	10^4^*Λ*_dEG_	*Λ*_EGd_	10^3^*Λ*_dca_	*Λ*_cad_	*Λ*_wEG_	*Λ*_EGw_	10^3^*Λ*_wca_	10^3^*Λ*_caw_	*Λ*_Egca_	*Λ*_caEG_	10^3^ RMSD (σ)
FLU (1) + water (2) + ChCl/MA (3)													
298.15	6.12	0.44	1.39	0.44	−0.01	0.02	0.09	7.18	0.01	0.01	−0.06	0.06	0.377
303.15	−1.90	0.21	6.48	0.22	−0.02	0.02	0.10	6.79	0.01	0.01	−0.06	0.07	0.608
308.15	6.89	−0.02	0.63	−0.97	−0.02	0.02	0.11	7.22	0.01	0.01	−0.06	0.07	0.977
313.15	0.02	−0.24	2.63	−0.21	−0.01	0.02	0.15	8.97	0.01	0.01	−0.06	0.05	0.487

The correlation parameters calculated using the e-NRTL model for FCZ solubility in aqueous DES solutions have been provided within Table 6.

The correlation parameters calculated using the Wilson model for FCZ solubility in aqueous DES solutions have been provided within Table 7.

### 3.5 Intrinsic fluorescence spectroscopy analysis

**Fig. 4:**
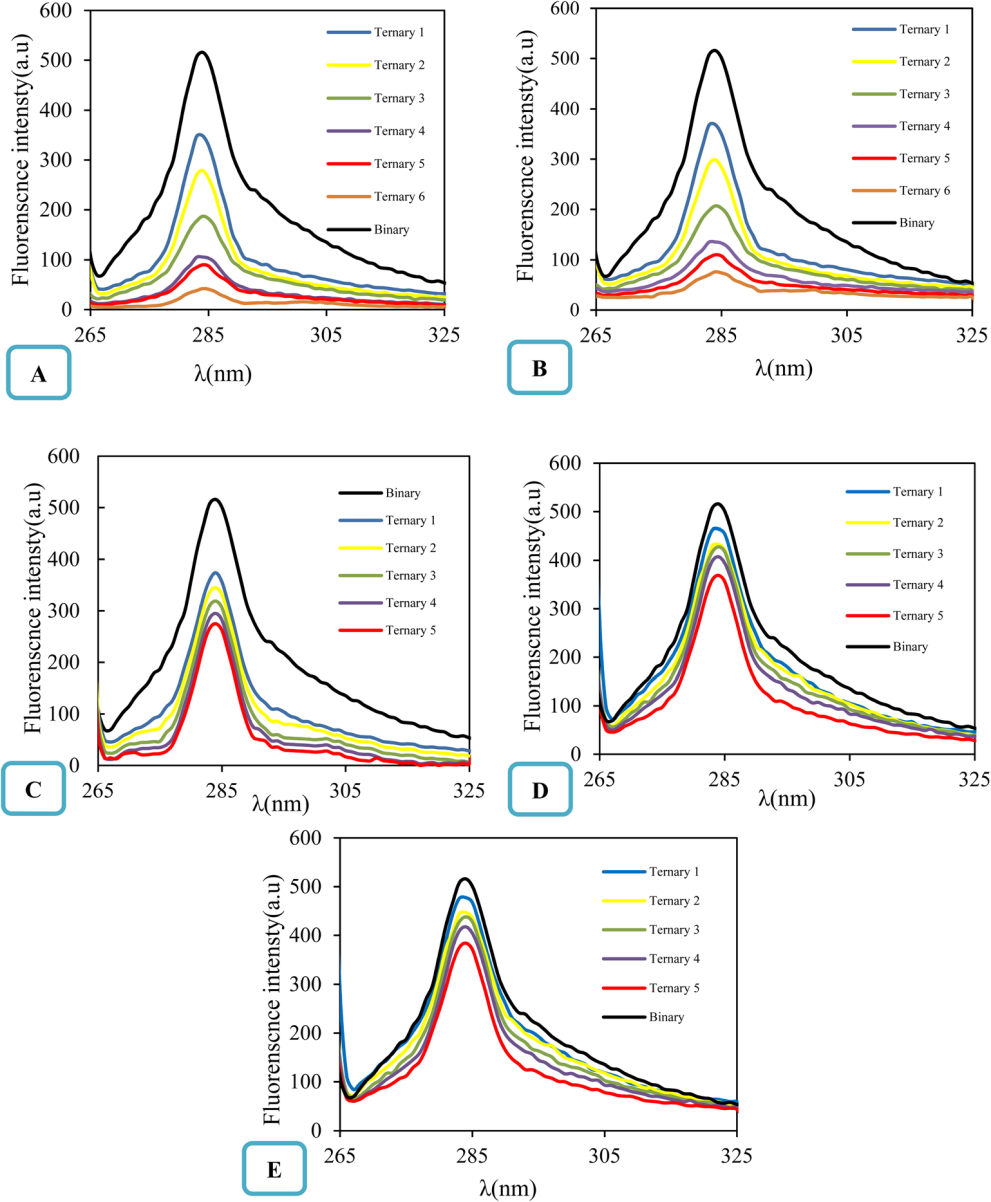
Fluorescence spectra of FCZ (20 μM) in presence of different DESs ((A) ChCl/OX with concentration of 0, 37, 55, 82, 110, 146, 182 μM; (B) ChCl/MA with concentration of 0, 36, 58, 85, 110, 150, 183 μM; (C) ChCl/EG with concentration of 0, 35, 56, 90, 112, 153 μM; (D) ChCl/G with concentration of 0, 35, 56, 90, 112, 153 μM; (E) ChCl/U with concentration of 0, 35, 56, 90, 112, 153 μM, respectively) at 298.15 K.

Intrinsic fluorescence spectroscopy is an analytical technique employed for assaying the binding complexation between ligands and macromolecules by recording molecular transitions from the excited state to the ground state. The quenchers such as many molecular organic compounds can reduce the fluorescence intensity. Thus, the quenching of fluorescence substances and the reduction in fluorescence emission are very useful indicators for the investigation of interactions and existence of binds between fluorescence substances and quenchers.^47,48^ In this work, the fluorescence spectroscopy method was used to investigate the interaction between FCZ as a fluorescence substance and different ChCl-based deep eutectic solvents as quenchers. The fluorescence emission spectra of FCZ at 298.15 K without and in the presence of different concentrations of each DES are shown in Fig. 4.

The maximum emission wavelength of fluconazole (FCZ) was observed at 280 nm, with excitation occurring at 260 nm. As illustrated in Fig. 4, the fluorescence intensity of FCZ decreased with an increase in the concentration of each deep eutectic solvent (DES). This observation suggests that FCZ may interact with the studied DESs.^49–52^ The fluorescence quenching trend of FCZ, in relation to its intrinsic fluorescence, follows the order:ChCl/OX > ChCl/MA > ChCl/EG > ChCl/G > ChCl/U.

This pattern indicates a particularly strong interaction between FCZ and the DES composed of choline chloride (ChCl) and oxalic acid (OX). Fluorescence quenching is generally understood as a process that leads to a reduction in the fluorescence intensity of a fluorophore due to various molecular interactions. These interactions can include molecular rearrangements, energy transfer, excited-state reactions, collision quenching, and the formation of ground-state complexes. Understanding the nature and mechanism of quenching requires distinguishing between the two main types: static and dynamic quenching. The mechanism by which the dynamic quenching occurs involves the interaction between the fluorophore (FCZ) and the quencher (DES) during their excited state. When these two entities collide, energy is dissipated, which subsequently diminishes the quantum yield and reduces fluorescence intensity, ultimately inhibiting fluorescence emission. In contrast, static quenching occurs when the quencher (DES) and the fluorophore (FCZ) form a non-fluorescent complex in the ground state, meaning the fluorophore never gets excited in the first place or its excited state cannot emit light.^53–56^ These quenching types are usually evaluated using the Stern–Volmer equation:^57^18
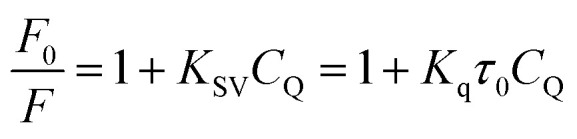
19
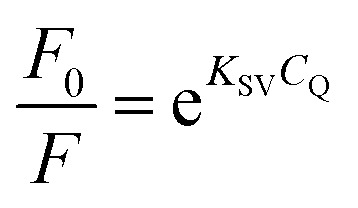
20
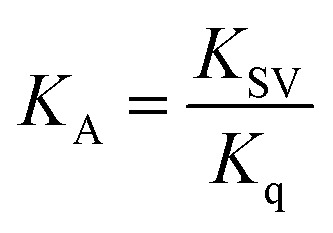
where *F*_0_ and *F* are the fluorescence intensities of fluorophore in the absence and presence of quenching agent, *C*_Q_ is the concentration of quenching agent, and *K*_SV_ is the Stern–Volmer quenching constant, which can be written as *K*_SV_ = *K*_q_*τ*_0_, where *K*_q_ is the quenching rate constant, *τ*_0_ is the average fluorescence lifetime of the drug in the absence of quencher. Eqn (18) and (19) are classic and modified Stern–Volmer equations, and they are used to analyze the fluorescence emission results by plotting the *F*_0_/*F* of fluorescence substance *versus* the concentration of quenching agent.^58^ The association constant (*K*_A_) presented in eqn (20), describes the equilibrium constant for the formation of a non-fluorescent complex between FCZ and the DES. A higher *K*_A_ indicates a more stable FCZ–DES complex, further supporting a static quenching mechanism.^59–62^

**Fig. 5:**
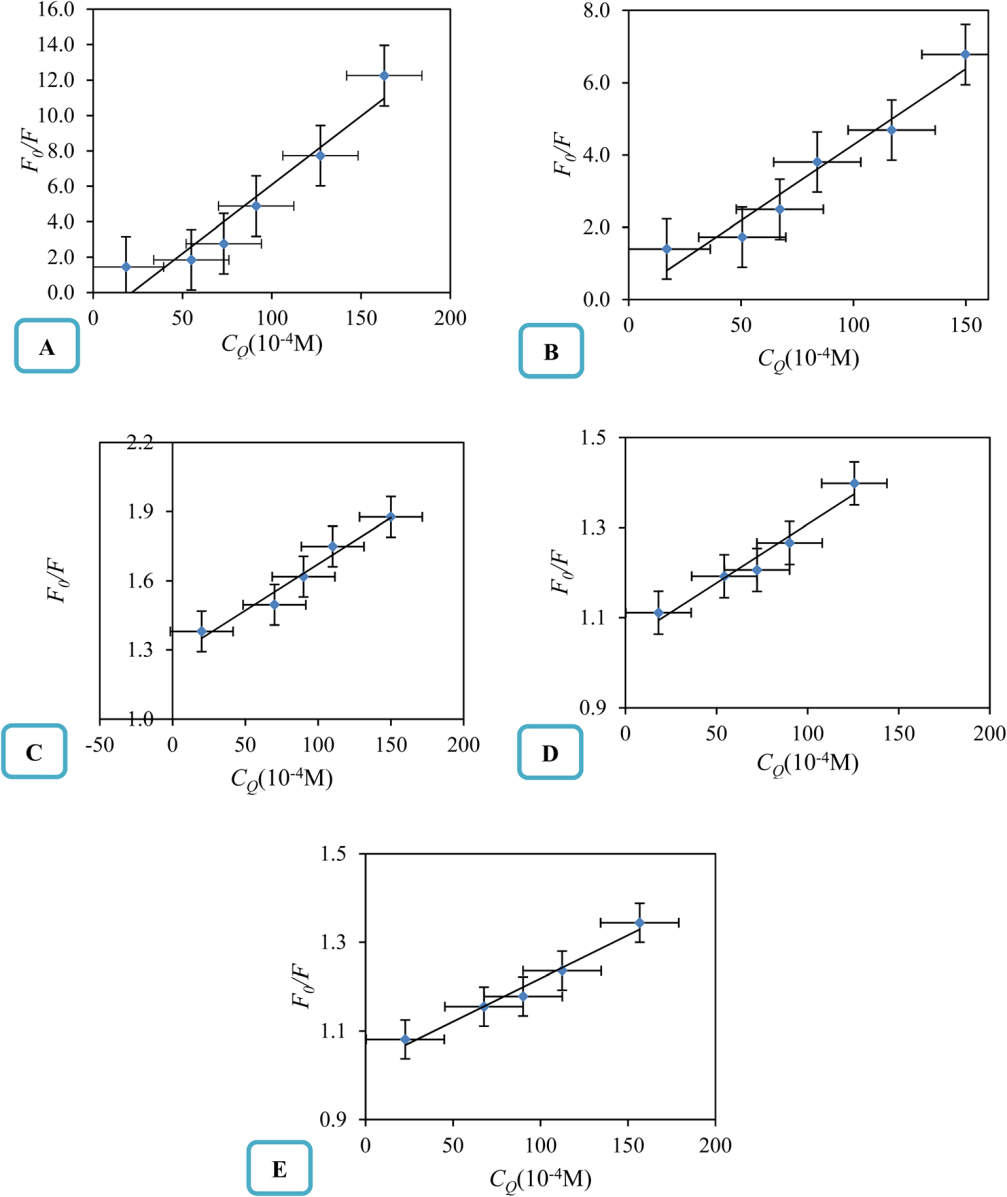
The Stern–Volmer plot of the FCZ-DESs ((A) ChCl/OX with concentration of 0, 37, 55, 82, 110, 146, 182 μM; (B) ChCl/MA with concentration of 0, 36, 58, 85, 110, 150, 183 μM; (C) ChCl/EG with concentration of 0, 35, 56, 90, 112, 153 μM; (D) ChCl/G with concentration of 0, 35, 56, 90, 112, 153 μM; (E) ChCl/U with concentration of 0, 35, 56, 90, 112, 153 μM, respectively) at 298.15 K.

The linearity of the Stern–Volmer plot, as described by eqn (18), may indicate the presence of a single binding site near the fluorophore for the quencher, which can result in either static or dynamic quenching. Conversely, the observation of an upward curvature in the plot suggests the involvement of multiple quenching mechanisms or the existence of additional binding sites. To quantitatively analyze such upward-curved Stern–Volmer plots, particularly those frequently encountered at higher concentrations, eqn (19) is employed.^63–68^ The Stern–Volmer plots for drug-deep eutectic solvent (DES) complexes are presented in Fig. 5.

From this depiction, it can be seen that the Stern–Volmer curves are linier for all the studied DESs and indicate that there is only one type of quenching for drug–DES complexes.^64,69^

The fluorescence quenching constants *K*_q_ and *K*_SV_ values of FCZ with all the studied DESs have been listed in Table 8. Generally, the maximum collisional quenching constant, *K*_q,r_, of different quenchers with the biopolymers was obtained about 2 × 10^10^ M^−1^ s. If the *K*_q_ > *K*_q,r_, the dynamic quenching certainly is not the reason of fluorescence quenching of biopolymers.^11,70^

**Table 8:** The quenching constants (*K*_sv_ and *K*_q_) and binding site number (*n*) of the interaction between fluconazole and different deep eutectic solvent at 298.15 K^a^^,^^b^

DESs	*K*_SV_ (10^4^ M^−1^)	*K*_q_ (10^12^ M^−1^ s^−1^)	*K*_A_ (10^4^ M^−1^)	*n*
ChCl/OX	14.92	1.49	1.20	4.90
ChCl/MA	12.53	1.25	1.12	2.86
ChCl/EG	2.45	0.24	1.03	0.84
ChCl/G	2.09	0.21	1.017	0.73
ChCl/U	1.62	0.16	1.016	0.68

a Standard uncertainty of temperature is *u*(*T*) = 0.1 K.

b The standard uncertainty, *u*_c_, for the dynamic quenching constants, *K*_SV_, bimolecular quenching constant, *K*_q_, and static quenching constant, *K*_A_, are *u*_c_ (*K*_SV_) = 0.06 × 10^−4^ M^−1^, *u*_c_ (*K*_q_) = 0.15 × 10^−12^ M^−1^ s^−1^, and *u*_c_ (*K*_A_) = 0.06 × 10^−4^ M^−1^, respectively.

In this work, the *K*_SV_ and *K*_q_ values for studied systems were obtained about 10^4^ M^−1^ and 10^12^ M^−1^ s, respectively. Obviously, the *K*_q_ value of drug quenching procedure in presence of each investigated DES are greater than the *K*_q,r_of the scattered procedure.^47^ These results indicate that the quenching is not initiated from dynamic collision and this is because of the formation of a complex between drug and DES.^71^

The quenching constants (*K*_sv_ and *K*_q_) listed in Table 6 are crucial for understanding the nature of the interactions between fluconazole (FCZ) and the different deep eutectic solvents (DESs). The dynamic quenching constant (*K*_q_) represents the collision rate between the excited-state FCZ and the DES components. The values observed for FCZ in the presence of different DESs are consistent with those typically reported for molecular collisions in fluorescence quenching studies. For example, the *K*_q_ values for ChCl/OX (14.92 × 10^9^ M^−1^ s^−1^) and ChCl/MA (12.53 × 10^9^ M^−1^ s^−1^) are comparable to those seen for dynamic quenching in other systems.^19,64,72^ The static quenching constant (*K*_sv_) reflects the formation of a non-fluorescent complex between FCZ and the DES. For example, the values for ChCl/OX (1.49 M^−1^) and ChCl/MA (1.25 M^−1^) suggest a significant static quenching contribution, which supports the hypothesis that these DESs interact with FCZ in a manner that leads to complex formation.^67,73–75^ The binding site numbers suggest the number of sites on FCZ that interact with the DES. The highest value (*n* = 4.90 for ChCl/OX) indicates a stronger interaction, and as expected, the binding site number decreases for other DESs in the order ChCl/OX > ChCl/MA > ChCl/EG > ChCl/G > ChCl/U. These constants are consistent with typical values found in literature for both dynamic and static quenching mechanisms.^19,76,77^ Additionally, the Stern–Volmer analysis and the observation of linear plots (Fig. 5) suggest that the quenching process in this study is predominantly governed by static quenching, with no significant contribution from dynamic collision quenching.

#### 3.5.1 Association constants and the number of binding sites

Assuming that the fluorescence quenching of the drug is a result of a static quenching process, where the quenching is attributed to the formation of a complex between FCZ and the quencher (DES), the equilibrium between the free and bound species can be approximated using the following equation:^11^21
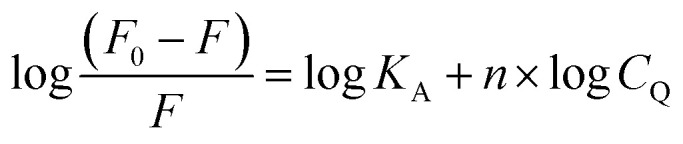
here *F*_0_, *F* and *C*_Q_ are the same as those in the Stern–Volmer equation. The association constant, *K*_A_, reflects the reaction extent of FCZ and quencher; The value of *n* represents the probable number of binding sites in FCZ, specifying the number of quenchers bound to a drug molecule. The values of *K*_A_ and *n* are calculated from the slope and the intercept of the 
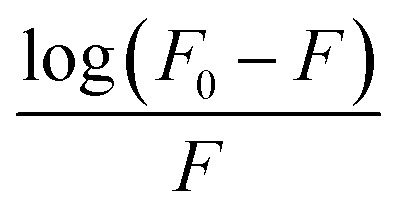
*versus* log *C*_Q_. These parameters are listed in Table 6. From the values of *n* for all the studied systems, it is inferred that the system containing FCZ in presence of DES mixed by ChCl/OX has a maximum binding side. For both the *K*_A_ and *n* values of FCZ-DES the observed trend is as follows: ChCl/OX > ChCl/MA > ChCl/EG > ChCl/G > ChCl/U. In other words, this trend shows that the interaction between FCZ and ChCl/OX is stronger than other studied DESs and FCZ-(ChCl/OX) complex is very stable. Also, these results can be related to the solubility of FCZ in presence of studied DEDs. This means that with increasing the *K*_A_ and *n* values of FCZ–DESs complexes the solubility of drug may be increased. There are many investigations about increasing the solubility of various drugs with ChCl-based DESs, especially the DESs used in this work.^18,26,78,79^

## 4. COSMO results

The theoretical framework of the study relies primarily on the DFT calculation on Dmol3 with COSMO results. Materials Studio (Biovia, 2023) employing the GGA VWN-BP functional was used to achieve the optimal results for the studied system, as recommended by the Dmol3 developers. Also, water was chosen as the solvent for the COSMO calculation. A two-step task including geometry and energy optimization GGA VWN-BP function, DND (3.5) basis set, and COSMO results. The COSMO results containing σ-profile illustrated in Fig. 7, and dielectric (solvation) energy and other properties that could be used for interpretation of hydration behavior of the studied chemicals besides the cavity surface area and volume that has presented in Table 9.

**Table 9:** The surface area, *A*, and total surface volume of cavity, *V*, dielectric (solvation) energy, HOMO and LUMO values and energies obtained from COSMO calculations

Chemicals	*V* (Å^3^)	*A* (Å^2^)	Dielectric (solvation) energy (kcal mol^−1^)	HOMO	LUMO	*E*_HOMO_	*E*_LUMO_
Fluconazole	304.383	276.228	−17.65	79	80	−6.310	−1.533
Choline chloride	174.229	185.441	−53.91	38	39	−5.043	0.420
Oxalic acid	86.495	105.740	−14.30	23	24	−6.633	−3.126
Malonic acid	107.268	126.257	−14.56	27	28	−6.436	−1.332
Ethylene glycol	77.527	97.835	−10.03	17	18	−6.226	1.148
Glycerol	106.944	126.093	−16.93	25	26	−6.095	1.242
Urea	69.415	89.217	−17.70	16	17	−5.994	0.641

Table 9, represents the surface cavity volumes of the individual molecules studied, where these volumes are indicative of the spatial distribution of the molecules and provide insights into their interaction potential. Surface cavity volume reflects the intensity of molecular interactions and is analogous to the apparent molar volume, which includes both the molecule's intrinsic volume and the surrounding voids influenced by solvation or intermolecular effects.^6,21,80–83^

In the context of the study, fluconazole is the drug of interest, and prior solubility investigations have demonstrated that fluconazole exhibits the highest solubility in a deep eutectic solvent (DES) composed of choline chloride and oxalic acid. However, since the DES itself is not included in the molecules analyzed in this illustration, the interpretation must be based solely on the provided individual components. From the representation of surface cavity volumes, fluconazole appears to have a larger and more complex molecular structure compared to smaller and less complex molecules such as ethylene glycol, glycerol, and urea. This larger surface cavity volume is consistent with fluconazole's higher molecular weight and the presence of multiple functional groups, such as aromatic rings and nitrogen-containing moieties. These features not only contribute to fluconazole's electron density distribution, as observed in its σ-profile, but also suggest a greater potential for interactions with solvents, particularly polar and hydrogen-bond-donating/accepting solvents.^18,21,80,84–86^ Choline chloride, with its quaternary ammonium group, and oxalic acid, with its highly electronegative carboxyl groups, are polar and capable of forming strong intermolecular interactions. While their surface cavity volumes are not explicitly compared in this depiction, their physicochemical properties suggest a strong affinity for fluconazole, which likely enhances solubility when they are combined in a DES. The solubility enhancement is likely driven by the DES's ability to engage fluconazole through hydrogen bonding, ionic interactions, and dipole–dipole interactions, facilitated by the complementary functional groups present in choline chloride and oxalic acid. For the other compounds in the Table 7, the relatively smaller surface cavity volumes of ethylene glycol, glycerol, and urea suggest more compact structures with fewer interaction sites, limiting their ability to dissolve larger, structurally complex molecules like fluconazole effectively.^87–89^

While oxalic acid and malonic acid contain carboxyl groups capable of significant interactions, their individual profiles do not achieve the synergistic solubilization effect observed in the DES with choline chloride. In conclusion, while fluconazole's solubility in the choline chloride/oxalic acid DES cannot be directly inferred from Table 7 due to the absence of the DES itself, the surface cavity volumes and structural features of the individual components help rationalize the molecular interactions underlying solubility trends. Fluconazole's large surface cavity and diverse functional groups make it particularly responsive to solvents that can engage in strong hydrogen bonding and polar interactions, properties exemplified by choline chloride and oxalic acid in a DES configuration.^18–20,87^

**Fig. 6:**
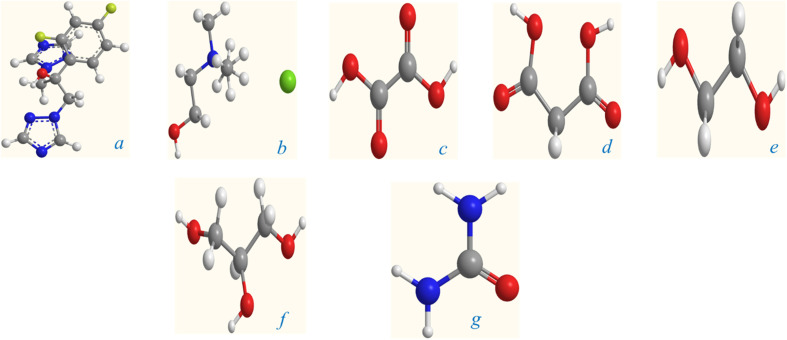
Optimized molecular structure of (a) Fluconazole, (b) choline chloride, (c) oxalic acid, (d) malonic acid, (e) ethylene glycol, (f) glycerol, (g) urea.

**Fig. 7:**
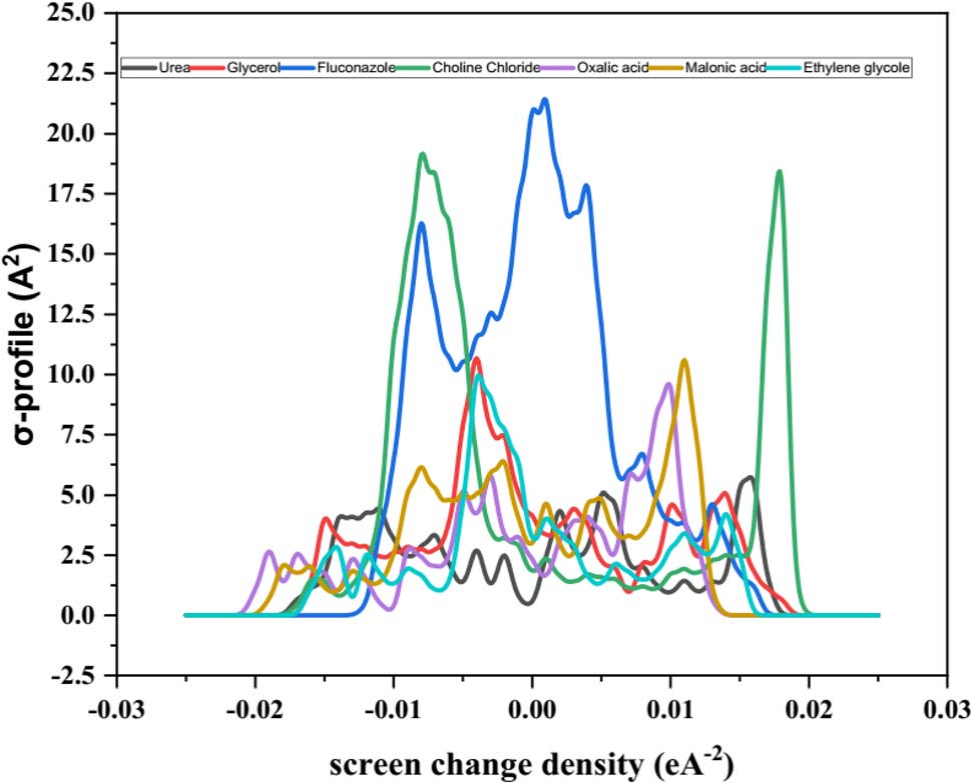
The σ-profile plots obtained from COSMO-DFT result.

The σ-profile analysis provides a powerful approach for understanding the electron density distribution within molecules, enabling insights into molecular reactivity, intermolecular interactions, and physicochemical properties.^87,90^ The σ-profile represents the screening charge density as a function of electron density regions across a molecule's surface. Peaks observed in these profiles correspond to areas of significant electron density, which are often associated with specific functional groups or structural features. The σ-profile is thus a useful tool for predicting a molecule's dipole moment and its interactions with other molecules, including solvents, ions, or charged species. In the analysis of the seven molecules presented (3D structure given in Fig. 6), the σ-profiles reveal key characteristics of their electron density distributions, as shown in Fig. 7. Most of these distributions display a predominant negative charge density, a feature commonly associated with ionic compounds or polar molecules, where substantial charge separation is observed. This is a characteristic that also aligns with the behavior of ionic liquids, where the significant difference in charge distribution between cations and anions contributes to their unique properties. The analysis demonstrates distinct trends for each compound, highlighting their molecular features and functional groups.^91–93^

The σ-profile of fluconazole exhibits a complex distribution characterized by multiple peaks. This complexity arises from the diverse functional groups present within its molecular structure, including aromatic rings and nitrogen-containing moieties. These structural elements contribute to the regions of high and varied electron density observed in the profile. Such complexity suggests that fluconazole possesses several reactive sites and can engage in multiple types of intermolecular interactions, such as hydrogen bonding or π–π stacking, depending on the surrounding environment.^94–96^

In contrast, the σ-profile of choline chloride is characterized by a more prominent single peak, indicative of a region with a high concentration of electron density. This observation corresponds to the presence of the quaternary ammonium group in the molecule, which is highly polarized. The quaternary ammonium cation carries a significant positive charge, and the accompanying electron density reflects the substantial contribution of this functional group to the molecule's overall profile. This relatively simple but distinct feature highlights the ionic nature of choline chloride and its potential interactions with anions or polar solvents.^77,91,97,98^

The dicarboxylic acids, oxalic acid and malonic acid, exhibit distinct σ-profiles with well-defined peaks that correspond to their carboxyl functional groups. These groups, which contain electronegative oxygen atoms, are known for their high electron density. The peaks in the σ-profiles of these acids reflect the significant contribution of these electronegative atoms to the molecule's charge distribution. The presence of two carboxyl groups in each molecule further amplifies the electron density in specific regions, distinguishing these acids from other compounds analyzed.^99^

The σ-profiles of ethylene glycol and glycerol are comparatively simpler, with fewer prominent peaks. This observation can be attributed to their smaller molecular sizes and less complex structures relative to other compounds in the analysis. Both molecules possess hydroxyl groups, which contribute to the electron density distribution, but their contributions are less pronounced compared to the carboxyl groups in dicarboxylic acids or the quaternary ammonium group in choline chloride. The sigma profiles of these molecules reflect their reduced molecular complexity and suggest fewer reactive sites.^100–102^

Urea also exhibits a relatively smooth σ-profile with fewer distinct peaks. Its simpler molecular structure, consisting primarily of a carbonyl group and amino groups, contributes to its modest electron density distribution. The absence of highly electronegative or structurally complex functional groups limits the variation in the σ-profile, making urea's distribution less intricate compared to fluconazole or the dicarboxylic acids.^103,104^

The σ-profiles presented in Fig. 7 highlight the unique electronic characteristics of each compound, reflecting their molecular structures and functional groups. The distribution of electron density, as evidenced by the peaks and their intensity, provides insights into the chemical behavior of these molecules. Peaks associated with specific functional groups, such as carboxyl groups, hydroxyl groups, aromatic rings, and nitrogen-containing moieties, reveal regions of potential reactivity and sites for intermolecular interactions. For instance, the complex profile of fluconazole suggests its potential for diverse reactivity and interaction mechanisms, whereas the more defined peaks in oxalic acid and malonic acid indicate the significant influence of carboxyl groups. The simpler profiles of ethylene glycol, glycerol, and urea reflect their less complex structures and fewer reactive sites. Overall, the σ-profile analysis demonstrates its utility in identifying and characterizing electron density distributions within molecules. By correlating these distributions with molecular structure and functional groups, valuable insights into a molecule's physicochemical properties, reactivity, and potential interactions can be obtained. This approach offers a robust framework for analyzing diverse chemical systems and understanding their behavior in various environments.^105–108^

## 5. Deeper analysis of molecular interactions and solubility enhancement

The solubility enhancement of fluconazole (FCZ) in deep eutectic solvents (DESs) observed in this study can be attributed to a complex interplay of intermolecular forces, including hydrogen bonding, van der Waals interactions, and ion–dipole forces. These forces not only dictate the overall solubility but also control the stability of the solute–solvent complexes formed within the DES matrix. The enhanced solubility of FCZ in DESs compared to aqueous solutions is primarily driven by the stronger and more diverse intermolecular interactions provided by the DES components, specifically choline chloride (ChCl) and oxalic acid. Hydrogen bonding plays a critical role in the solubility of FCZ in DESs. Both FCZ and the individual components of the DES ChCl, oxalic acid, and others possess functional groups capable of hydrogen bond formation. FCZ contains hydroxyl (–OH) and amino (–NH) groups, while the DES components such as oxalic acid and glycerol also have hydroxyl and carboxyl (–COOH) groups. These functional groups facilitate the formation of solute–solvent complexes, with the hydroxyl groups of FCZ and oxalic acid forming hydrogen bonds with each other. The strength and nature of these hydrogen bonds are crucial for the solubility enhancement of FCZ, as they provide a strong, stable interaction between the drug and the solvent.^35,109–111^

The COSMO calculations (using DFT with the GGA VWN-BP functional) help quantify these interactions. The σ-profiles obtained from the COSMO results provide detailed insights into the electron density distribution on the molecular surfaces of the individual components. The σ-profile of FCZ (shown in Fig. 7) reveals a complex distribution of electron density, particularly around the hydroxyl and amino groups, which are likely involved in hydrogen bonding interactions. These high electron density regions are ideal for interacting with the hydrogen bond donor sites in the DES, particularly the hydroxyl groups of choline chloride and oxalic acid.^6,19,86,109^

In addition to hydrogen bonding, ion–dipole interactions play a significant role in the solubility behavior of FCZ in DESs. Choline chloride, with its quaternary ammonium cation (ChCl^+^), and oxalic acid, with its carboxyl anion (C_2_H_2_O_4_^2−^), introduce strong ion–dipole interactions. These interactions occur when the polar FCZ molecule, with its electronegative oxygen and nitrogen atoms, interacts with the charged components of the DES. The σ-profile of ChCl (shown in Fig. 7) exhibits a prominent positive charge distribution, particularly around the quaternary ammonium group. This charge distribution reflects the strong dipole character of ChCl, which can interact with the negatively charged regions of FCZ. This ion–dipole interaction is crucial in stabilizing the solute–solvent complex and contributes to the observed solubility enhancement. Similarly, the carboxyl groups in oxalic acid have a negative charge distribution, which interacts with the positively polarized regions of FCZ, particularly its aromatic rings and nitrogen-containing groups. The COSMO analysis also highlights the role of the dielectric (solvation) energy in these ion–dipole interactions. The dielectric solvation energy for ChCl is notably high at −53.91 kcal mol^−1^, as presented in Table 9. This energy reflects the strong electrostatic interactions between the ChCl ions and FCZ, further stabilizing the drug–solvent complex. In addition to hydrogen bonding and ion–dipole interactions, van der Waals forces, particularly dipole–dipole interactions, also contribute to the overall solvation process. These interactions arise from the temporary fluctuations in electron density that create transient dipoles. The σ-profile of FCZ shows multiple peaks, particularly around its aromatic rings and nitrogen-containing moieties, which indicates the presence of sites with significant electron density that can interact *via* dipole–dipole forces. These weak, attractive forces enhance the stability of the solute–solvent complex and aid in the overall solvation of FCZ in DESs. The σ-profiles of ethylene glycol, glycerol, and urea are simpler, with fewer prominent peaks, indicating fewer reactive sites and lower potential for significant dipole–dipole interactions. These molecules possess fewer sites for hydrogen bonding or ion–dipole interactions, making them less effective in enhancing the solubility of FCZ compared to more complex DES components like ChCl and oxalic acid. The surface cavity volumes and dielectric (solvation) energy obtained from the COSMO calculations provide additional quantitative support for the claims made about these molecular interactions. Table 9 summarizes the surface area and volume of the cavity, which reflects the spatial distribution of the molecules and their interaction potential. The larger surface cavity volume of FCZ (304.383 Å^3^) compared to the smaller volumes of the other molecules such as urea (69.415 Å^3^) and ethylene glycol (77.527 Å^3^) indicates that FCZ has more room to form multiple interactions with the DES components. This larger cavity volume corresponds to a greater number of potential interaction sites for hydrogen bonding, ion–dipole, and van der Waals forces, which all contribute to the enhanced solubility. Additionally, the dielectric energy for ChCl (−53.91 kcal mol^−1^) and oxalic acid (−14.30 kcal mol^−1^) indicates their strong solvating ability, further supporting the idea that ion–dipole interactions are crucial in enhancing FCZ's solubility within the DES. These values suggest that ChCl, with its high dielectric energy, is particularly effective at stabilizing FCZ through electrostatic interactions. Fig. 7 provides the σ-profiles of the various molecules, showing their electron density distributions. These profiles are particularly useful for visualizing the regions of high electron density that are available for intermolecular interactions. For instance, the σ-profile of FCZ shows peaks around its hydroxyl and nitrogen groups, which are likely to participate in hydrogen bonding and ion–dipole interactions. The σ-profile of ChCl shows a strong positive peak corresponding to its quaternary ammonium group, which aligns with the regions of negative charge density on FCZ, indicating the potential for strong ion–dipole interactions. In conclusion, the enhanced solubility of fluconazole in DESs can be explained by the combined effects of hydrogen bonding, ion–dipole interactions, and van der Waals forces. The COSMO results, particularly the σ-profile, surface cavity volumes, and dielectric energy values, provide quantitative and visual support for these claims, offering a deeper understanding of the molecular interactions that govern the solubility of FCZ in DESs. The ion–dipole interactions, in particular, are shown to play a crucial role in stabilizing the drug–solvent complex, with ChCl and oxalic acid providing a highly favorable solvation environment for FCZ compared to other solvents.

## 6. Conclusion

This study provides a comprehensive investigation into the interactions between fluconazole (FCZ) and various deep eutectic solvents (DESs), formed by choline chloride (ChCl) as the hydrogen bond acceptor (HBA) and a range of hydrogen bond donors (HBDs) such as oxalic acid, malonic acid, ethylene glycol, glycerol, and urea. Fluorescence spectroscopy, conducted at a temperature of 298.15 K, was used to analyze the fluorescence quenching of FCZ in the presence of these DESs. The study found that the fluorescence quenching followed a static quenching mechanism, indicating that the interaction between FCZ and DESs resulted from the formation of a molecular complex rather than from dynamic collisions. This observation was further supported by the calculation of the Stern–Volmer quenching constant (*K*_q_) the quenching rate constant (*K*_SV_), which confirmed that the quenching was not initiated by dynamic collision events but rather by the formation of stable complexes between FCZ and the DES components.

The analysis also led to the determination of the association constants (*K*_A_) and the number of binding sites (*n*) for the fluconazole-DES systems. Among the DESs studied, the ChCl/oxalic acid system exhibited the highest binding affinity, with FCZ showing the strongest interaction with this particular DES. This result suggests that the ChCl/oxalic acid DES forms a more stable complex with FCZ compared to the other DES systems, highlighting the importance of specific molecular interactions in determining the solubility enhancement of FCZ. Solubility measurements for FCZ in the presence of these DESs were performed at varying DES weight fractions and temperatures. The study found that the solubility of FCZ increased with both the DES weight fraction and temperature, indicating a favorable solubility behavior in these systems. Among the various DESs tested, the ChCl/oxalic acid mixture demonstrated the highest solubility enhancement for FCZ, making it an ideal candidate for improving the solubility of this drug. This finding is crucial for the pharmaceutical industry, where solubility is often a limiting factor in drug bioavailability. To further understand the solubility behavior, the Wilson and e-NRTL models were employed to correlate the experimental solubility data. The results indicated that the Wilson model provided a superior fit for the solubility data in aqueous solutions, outperforming the e-NRTL model in this case. This suggests that the Wilson model is more suitable for describing the thermodynamic interactions in the FCZ–DES systems, providing a deeper understanding of the underlying solubility mechanisms. The thermodynamic analysis of the dissolution process in these DES systems revealed that all the DES systems exhibited endothermic and entropy-driven dissolution behavior. Positive values for both enthalpy and entropy indicated that the dissolution of FCZ in these DESs is thermodynamically favorable, and that the process is primarily driven by an increase in entropy. This result supports the notion that the DES systems enhance the solubility of FCZ through favorable thermodynamic conditions, which could lead to improved drug formulation strategies.

From a computational perspective, density functional theory (DFT) calculations with the COSMO solvation model were utilized to provide a deeper insight into the molecular interactions between FCZ and the DESs. The analysis of surface cavity volumes, dielectric energy, and σ-profiles revealed significant details about the molecular characteristics of FCZ and the individual components of the DESs, such as choline chloride and oxalic acid. The results showed that FCZ, with its large surface cavity and functional groups, has a strong propensity for solvation in polar and hydrogen-bonding solvents like choline chloride and oxalic acid, which together form a DES with enhanced solubility properties. The σ-profile analysis further revealed the distinct electron density distributions of the studied compounds, underscoring their potential for various intermolecular interactions that govern solubility behavior. The findings of this study contribute valuable insights into the molecular-level interactions and solubility behavior of fluconazole in DESs. The study also lays the groundwork for future investigations into similar drug–solvent systems, offering new strategies for the enhancement of drug solubility and bioavailability using DESs. The broader impact of this study lies in the sustainability potential of deep eutectic solvents (DESs) and their contribution to the principles of green chemistry. DESs, which are typically composed of natural, non-toxic, and biodegradable components such as choline chloride and organic acids or alcohols, provide a promising alternative to conventional solvents. Traditional organic solvents, often used in pharmaceutical and chemical industries, are frequently volatile, toxic, and harmful to both human health and the environment. In contrast, DESs are considered “green solvents” due to their low environmental impact, non-toxicity, and the ability to be synthesized from renewable resources. This study demonstrates that DESs, particularly the ChCl/oxalic acid system, not only improve the solubility of pharmaceutical compounds such as fluconazole but also align with green chemistry principles. The use of DESs in drug solubility enhancement represents a sustainable approach that reduces the reliance on harmful organic solvents and minimizes the environmental footprint of pharmaceutical manufacturing processes. The ability to tailor DESs for specific solubility and interaction profiles further supports their versatility and utility in a wide range of industrial applications, including drug formulation, biocatalysis, and chemical synthesis. Moreover, the enhanced solubility and stability of fluconazole in DESs could lead to more effective drug formulations, improving the bioavailability and therapeutic efficacy of poorly soluble drugs. This, in turn, could help reduce the dosage required for effective treatment, minimizing waste and reducing the environmental burden associated with pharmaceutical production. In conclusion, this study highlights the significant role that DESs can play in promoting sustainability in the pharmaceutical industry by offering eco-friendly, effective alternatives to traditional solvents. The findings support the growing body of evidence advocating for the use of DESs in green chemistry, reinforcing their potential to contribute to more sustainable, efficient, and environmentally conscious chemical processes.

## Data availability

The authors confirm that the data supporting the findings of this study are available within the manuscript, figures, and tables.

## Conflicts of interest

The authors declare no competing interests.
